# Direct and indirect mechanisms of KLK4 inhibition revealed by structure and dynamics

**DOI:** 10.1038/srep35385

**Published:** 2016-10-21

**Authors:** Blake T. Riley, Olga Ilyichova, Mauricio G. S. Costa, Benjamin T. Porebski, Simon J. de Veer, Joakim E. Swedberg, Itamar Kass, Jonathan M. Harris, David E. Hoke, Ashley M. Buckle

**Affiliations:** 1 Department of Biochemistry and Molecular Biology, Biomedicine Discovery Institute, Monash University, Clayton, Victoria 3800, Australia; 2 Programa de Computação Científica, Fundação Oswaldo Cruz, Rio de Janeiro, Brazil; 3 Institute of Health and Biomedical Innovation, Queensland University of Technology, Brisbane, Queensland 4059, Australia; 4 Institute for Molecular Bioscience, The University of Queensland, Brisbane, QLD 4072, Australia

## Abstract

The kallikrein-related peptidase (KLK) family of proteases is involved in many aspects of human health and disease. One member of this family, KLK4, has been implicated in cancer development and metastasis. Understanding mechanisms of inactivation are critical to developing selective KLK4 inhibitors. We have determined the X-ray crystal structures of KLK4 in complex with both sunflower trypsin inhibitor-1 (SFTI-1) and a rationally designed SFTI-1 derivative to atomic (~1 Å) resolution, as well as with bound nickel. These structures offer a structural rationalization for the potency and selectivity of these inhibitors, and together with MD simulation and computational analysis, reveal a dynamic pathway between the metal binding exosite and the active site, providing key details of a previously proposed allosteric mode of inhibition. Collectively, this work provides insight into both direct and indirect mechanisms of inhibition for KLK4 that have broad implications for the enzymology of the serine protease superfamily, and may potentially be exploited for the design of therapeutic inhibitors.

The kallikrein (*KLK*) gene family is the largest protease gene cluster in the human genome and encodes 15 serine proteases that have sequence identity varying from 40–80%, with a high degree of structural similarity around the active site^1^. The KLK proteases are involved in development and normal physiology but have also been implicated in cancer progression^2^. KLK4 is predominantly expressed in basal and secretory cells of the prostate gland with lower levels of expression in a number of tissues including breast, ovaries, thyroid, testis and developing teeth^3^,^4^,^5^. Over-expression of KLK4 has been documented in malignant prostate, ovarian and breast tumors and is associated with metastasis^6^,^7^,^8^, and mechanisms underpinning resistance to androgen deprivation therapy^9^. Conversely, KLK4 inhibition has resulted in reduced proliferation and spheroid formation in tissue culture based systems^10^. Therefore, developing specific ways of inhibiting KLK4 offers an attractive route for future therapeutic strategies.

The most direct way to inhibit KLK4 is by designing specific active site inhibitors. The Bowman-Birk family of protease inhibitors (Bowman-Birk Inhibitors (BBI)) are a class of proteins and peptides found in plants and plant seeds, where they are thought to play a role in defense against pathogens^11^. The smallest BBI described to date is sunflower trypsin inhibitor-1 (SFTI-1)^12^, which has also been found to inhibit other serine proteases, including KLK4^13^. SFTI-1 is a 14 amino acid bicyclic peptide (1GR**C**TKSIPPI**C**FPD14). The backbone cyclization coupled with an intramolecular disulfide linkage causes SFTI-1 to adopt a constrained structure in solution^14^. SFTI-1 binds trypsin by mimicking a substrate, where Lys5 inserts into the active site S_1_ specificity pocket^12^. Once bound, SFTI-1 can be cleaved, but the constrained nature of the SFTI-1 backbone maintains the same structure as the original uncleaved bound form, so it is not released. Rather, the bond is re-ligated and exists in equilibrium with the cleaved form, thus maintaining occupancy in the active site and prolonged inhibition. This mechanism conforms to the principles as outlined by Laskowski and is termed the ‘standard’ mechanism of protease inhibition^15^,^16^. The unique structural and functional properties of SFTI-1 make this backbone an ideal scaffold upon which new selectivities can be generated^17^.

SFTI-1 has been shown to inhibit KLK4 in addition to trypsin and matriptase^12^,^18^. Since the trypsin-SFTI-1 and matriptase-SFTI-1 protease/inhibitor complexes have been characterized by X-ray crystallography, a structural context is available to dissect relative potencies^12^,^19^. This information has been used to elucidate how a re-engineered SFTI-1 has reduced potency for trypsin and matriptase while gaining a 28-fold increased potency for KLK4^13^.

Allostery offers another route to KLK4 inhibition. KLK4, like the majority of serine proteases, is tightly regulated by conformational switches. Serine proteases are synthesized in an inactive zymogen state that has a distorted active site unable to efficiently support catalysis^20^. Upon cleavage of the propeptide, the new N-terminal isoleucine/leucine forms a salt bridge with Asp194 (chymotrypsin numbering used throughout the paper) to rigidify the oxyanion hole and active site catalytic triad. While most proteases are fully activated upon cleavage of the zymogen, some proteases use additional conformational switches such as ions or protein binding partners^21^,^22^ that also alter the rigidity of the active site. The Ile16 residue of KLK4 was previously shown to be released upon incubation with metals, consistent with an inhibitory role based upon formation of a destabilized, zymogen-like active site^23^. Since the metal-binding and active sites are separated by more than 20 Å, an allosteric mode of inhibition has been proposed but is not understood.

In order to investigate further both direct and indirect mechanisms of KLK4 inhibition here we describe the structures of KLK4 in complex with SFTI-1, an engineered SFTI-1 variant, and with nickel (KLK4-Ni). The very high resolution (~1.0 Å) of the KLK4-SFTI-1 structures provides exquisite detail of KLK4-inhibitor interactions and a structural rationalization for inhibitor potency and selectivity. Analysis of the KLK4-Ni structure reveals conformational heterogeneity, as well as metals bound at the active site and at an indirect inhibitory site. Combining this structure with molecular dynamics simulations depicts an indirect mechanism of information transfer resulting in active site inhibition. Collectively, this work provides insight into direct and indirect inhibition of KLK4 that has broad implications for the enzymology of serine proteases.

## Results and Discussion

### Structural rationalization of direct KLK4 inhibition by SFTI-1

**Figure 1:**
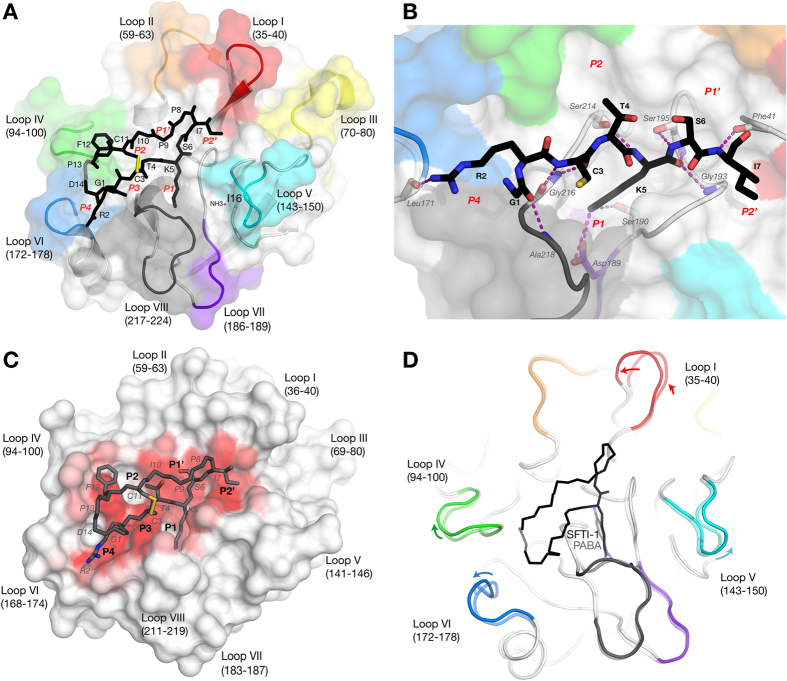
Overview of the KLK4-SFTI-1 interaction. (**A**) KLK4 with SFTI-1 bound in the active site. The backbone and interacting sidechains of SFTI-1 in the active site are shown as black sticks. KLK4 loops I-VIII are highlighted and labelled. Modelled SFTI atoms with missing density are transparent; (**B**) Close-up of KLK4-SFTI-1 interactions. KLK4 Salt-bridges and H-bonds are shown as broken magenta lines; (**C**) Surface of KLK4, colored by percentage surface burial upon SFTI-1 interaction (white-red gradient). Interacting SFTI-1 sidechains are shown as sticks; (**D**) Structural alignment of KLK4-SFTI-1 with KLK4-PABA^23^; PDB: 2BDG), highlighting differences.

We previously showed that SFTI-1 potently inhibits KLK4 proteolytic activity^13^,^24^. To examine the interaction between KLK4 and inhibitor we determined the X-ray crystal structure of a KLK4-SFTI-1 complex to 1.0 Å resolution. The inhibitor structure is well-resolved except for missing density in the backbone of Pro13 and the side chain of Asp14. The contact β-sheet of SFTI-1 has multiple interactions with the KLK4 active site, including the P1 Lys5 inserting into the S1 pocket of KLK4 (Fig. 1B).

Interface interactions are dominated by only 4 SFTI-1 residues: P1 Lys5, P4 Arg2, P2′ Ile7, and Phe12 (Fig. S1A & Table S1). Calculating the accessible surface area per residue that is buried upon interaction shows high complementarity between P4-P1′ (Fig. 1C & Table S1). SFTI-1 engages in 10 H-bonds and 2 salt bridges with KLK4 (Fig. 1B & Table S1). 8 of the H-bonds are mediated by the backbone of SFTI-1. The P1 residue Lys5 is the anchor residue since it engages in 4 H-bonds and 2 salt bridges, with its side-chain engaging in 1 out of 5 H-bonds and both salt bridges. The only other side-chain interaction is via Arg2. The interaction between protease and substrate-like inhibitors is mediated by 8 surrounding loops that are present in all S1 family proteases, dictating substrate specificity (Fig. 1A)^25^. The KLK4 buried surface area in the KLK4-SFTI-1 structure is contributed by all loops except loop III (Fig. 1C & S1B).

### Conformational changes upon binding SFTI-1

**Figure 2:**
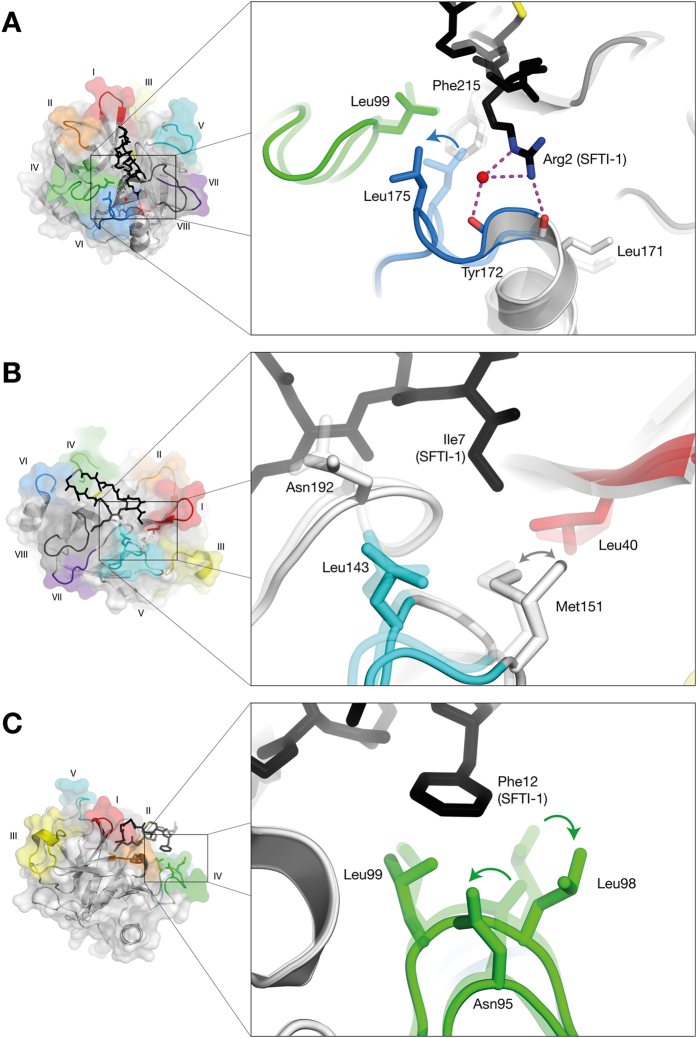
KLK4-SFTI-1 interactions. (**A**) Comparison of S4 subsites in KLK4-PABA (transparent) and KLK4-SFTI-1 (solid). SFTI bonds are shown as black sticks, H-bonds as broken lines, water molecules as red spheres; (**B**) Interactions at the S2′ site. The two rotamers of Met151 are shown; (**C**) SFTI-1 Phe12 is associated with a shift in KLK4 loop IV.

Overall, structural changes upon SFTI-1 binding are relatively minor when compared to the structure of KLK4 complexed with the inhibitor *p*-aminobenzamidine (PABA; PDB: 2BDG^23^; Root Mean Square Deviation (RMSD) = 0.345 Å and 0.338 Å for all backbone atoms, and backbone atoms of residues within 5 Å of SFTI-1, respectively; Fig. 1D). However, some specific active site changes are seen. Met151 (loop V), adopts two rotamers that both interact with the sidechain of P2′ Ile7 (Fig. 2B). To enable the hydrophobic environment of the S4 pocket to accommodate the positively charged P4 Arg, both loop VI Leu175 and the adjacent helix Leu171 shift, accompanied by recruitment of a water molecule that makes bridging H-bonds between P4 Arg and Tyr172 (loop VI) (Fig. 2A). Outside of the P4-P2′ residues, Phe12 which contributes 12% of the SFTI-1 buried surface area (Fig. S1A), induces changes in the conformations of loop IV (Fig. 2C). Notably, loop IV residues Asn95, Leu98 and Leu99 all adopt new side chain rotamers (Fig. 2C). These structural differences are indicative of conformational selection/induced fit upon SFTI-1 binding.

### SFTI-1 is a better fit for trypsin and matriptase than KLK4

**Figure 3:**
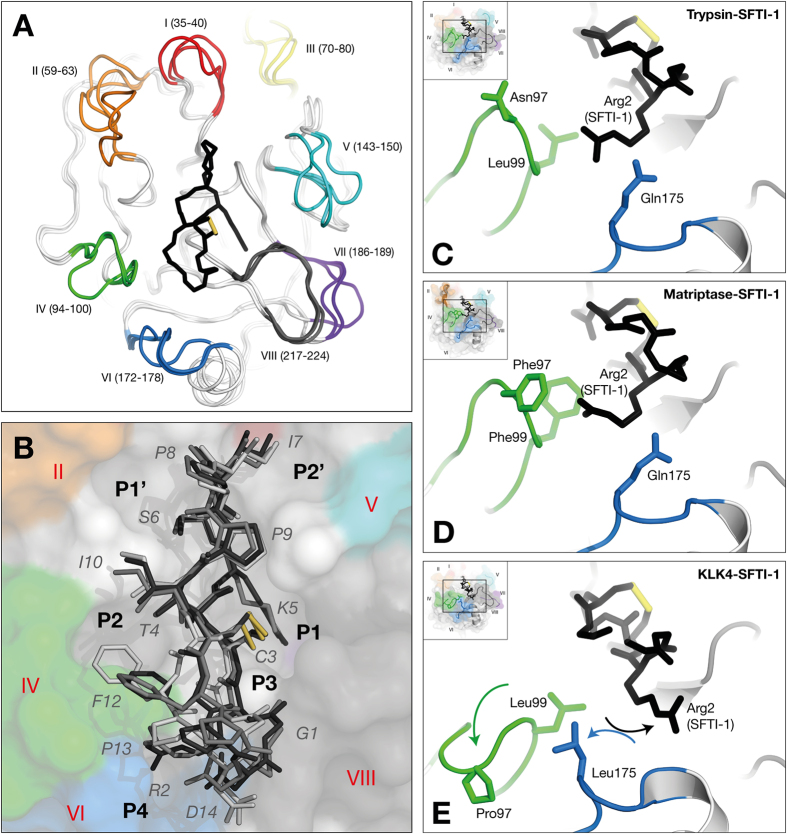
Comparison of protease-SFTI-1 complexes for trypsin, matriptase and KLK4. (**A**) Structural overlay of proteases. SFTI-1 backbone and P1 sidechain is shown in the active site for context. (**B**) Overlay of SFTI-1 (trypsin-SFTI-1 = charcoal, matriptase-SFTI-1 = grey, KLK4-SFTI-1 = silver) showing KLK4 surface for context. Close-ups of the S4 interactions with (**C**) trypsin, (**D**) matriptase, and (**E**) KLK4. In both trypsin and matriptase, Arg2 of SFTI-1 interacts with the backbone of loop IV. In KLK4, loop IV occupies a different (folded-over) conformation, Leu175 of loop VI shifts towards loop IV, and so the major interaction of Arg2 is with loop VI.

When comparing the structure of SFTI-1 in the context of trypsin, matriptase and KLK4, differences in the interface correlate to the potency of inhibition. While there is a discrepancy in the various SFTI-1 inhibition constants reported^12^,^26^,^27^, KLK4 is thought to be the least potently inhibited^13^. Comparison of the structures provides arguments to support this lowered inhibition. SFTI-1 has less buried surface area and fewer interactions with KLK4 than with either trypsin or matriptase (Table S1). SFTI-1 behaves as a Laskowski inhibitor, interacting with its targets following the classical lock-and-key model^14^,^17^. Indeed, for both trypsin^12^,^28^ and matriptase^19^,^29^,^30^ the final bound structures of inhibitor and protease each resemble the unbound states. In contrast, KLK4 undergoes significant conformational change at the P4/S4 site to accommodate SFTI-1 (Fig. 2A). A structural comparison of SFTI-1 complexed to KLK4, trypsin and matriptase (overall comparison between the three protein-SFTI complexes is shown in Fig. 3A) shows that one cycle of the bicyclic SFTI-1 aligns well (Thr4-Ile10) while the other half shows differences (Fig. 3B). Pro13 and the carboxylate of Asp14 have missing density in the KLK4 structure, yet are well-resolved in the context of trypsin and matriptase, showing evidence of mobility in the bound state. Second, in the KLK4 complex, rotation of the P4 Arg2 sidechain shifts its terminal guanidino group more than 7 Å relative to the other two complexes (Fig. 3C–E). This P4 Arg2 side chain movement in the context of KLK4 is associated with movements in loop VI (Fig. 2A). Taken together, decreased buried surface area, fewer interactions, and conformational adjustment of the protease^31^ are all consistent with a decreased inhibition of KLK4 compared to trypsin and matriptase.

### Rationalization of potency of SFTI-1_FCQR_ derivative

**Figure 4:**
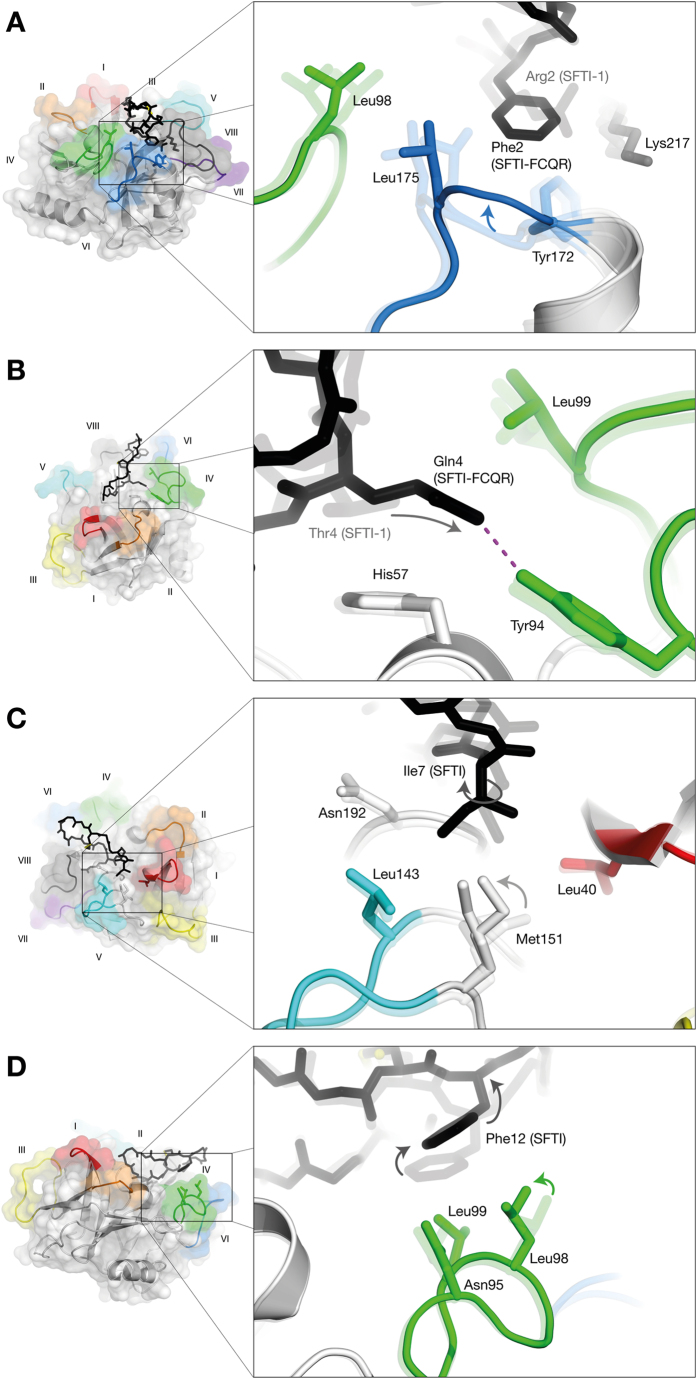
KLK4-SFTI-1_FCQR_ interactions. (**A**) P4 Phe at the S4 site is concomitant with a shift in the backbone of loop VI (solid), towards the inhibitor, compared to when S4 is empty (KLK4-PABA, transparent), or paired with Arg2 (KLK4-SFTI-1, transparent); (**B**) Mutation of P2 Thr4 (KLK4-SFTI-1, transparent) to Gln4 (KLK4-SFTI-1_FCQR_, solid) results in an increased buried surface area and allows a H-bond (broken line) with Tyr94. The conformation of loop IV is invariant across all structures, including KLK4-PABA (transparent); (**C**) P2′ Ile7 occupies different rotamers in KLK4-SFTI-1_FCQR_ (solid) and KLK4-SFTI-1 (transparent). The presence of two rotamers of Met151, in both KLK4-SFTI-1 and KLK4-SFTI-1_FCQR_, indicate conformational frustration at the S2′ site; (**D**) Phe12 shifts more than 2 Å compared with KLK4-SFTI-1 (transparent), and is associated with a lower buried surface area for Phe12 in the SFTI-1_FCQR_ structure. To accommodate the high burial in KLK4-SFTI-1 (transparent), Leu98 is displaced by 1.8 Å from the unoccupied KLK4-PABA conformation (not shown). It resumes the unoccupied conformation in KLK4-SFTI-1_FCQR_ (solid).

We previously tested the amidolytic activity of KLK4 against a focused library of synthetic tetrapeptide substrates with an invariant P1 Arg^13^. KLK4 displayed the highest activity for the FVQR-*p*-nitroanilide (*p*NA) substrate, with P4 and P2 residues affecting activity the most^13^. This sequence was grafted onto the SFTI-1 scaffold with the retention of the cysteine, creating SFTI-1_FCQR_ (SFTI-1 substituted with Phe2, Gln4 and Arg5), and resulted in a 18-fold increased inhibition of KLK4 (*K*_*i*_ = 3.59 nM)^13^. To investigate the structural basis for increased potency, we solved the crystal structure of a KLK4-SFTI-1_FCQR_ complex to 1.3 Å resolution. Compared with KLK4-SFTI-1 there is an overall increase in the buried surface area and number of protease-inhibitor interactions (Table S3 & Fig. S2), consistent with the increased inhibitory potency of SFTI-1_FCQR_. Binding can be mainly attributed to improved interactions at mutated residues P4, P2 and P1 (Fig. 4). At the S4 site, the KLK4-SFTI-1_FCQR_ structure shows conformational selection and/or induced fit upon binding, where the replacement of Arg at P4 with Phe is associated with a movement of loop VI towards the inhibitor, suggesting an improved interaction (Fig. 4A). Deep in the S2 pocket, the KLK4-SFTI-1_FCQR_ structure shows an interaction between Gln4^NE^ of SFTI-1_FCQR_ and Tyr94O of KLK4 (Fig. 4B). While there is still a requirement for hydrophobic interactions in the shallow of the S2 pocket, this observation provides a structural explanation for why the polarity of Gln is desirable in the S2 pocket, and goes some way to explaining the preference for Gln>Val>Leu>Thr at the S2 subsite previously reported^13^. There are also several secondary effects that contribute to improved binding at unchanged SFTI-1 residues, notably P2′ (Fig. 4C & S2, Table S3) and Phe12 (Fig. 4D & S2, Table S3). These secondary effects are seen as unbroken density for Pro13 and Asp14, which can now be fully resolved, in contrast to KLK4-SFTI-1. Therefore, increased potency of SFTI-1_FCQR_ can be rationalized by improvements in binding interactions coupled with favourable conformational changes.

### Structure of KLK4-Ni complex reveals previously hidden dynamics

**Figure 5:**
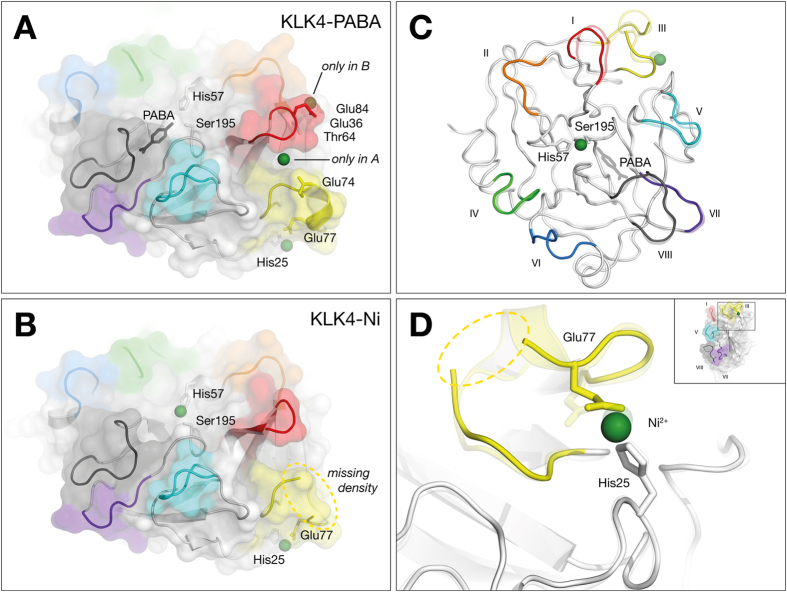
Structural comparison of KLK4 crystallized in the presence of 10mM nickel, (**A**) with 20 mM PABA (KLK4-PABA, 2BDG chains A and B) or (**B**) without PABA (KLK4-Ni). KLK4 is drawn as cartoon and surface. S1 pocket is occupied by PABA (sticks) in KLK4-PABA, and a Ni^2+^ ion (green sphere) in KLK4-Ni, interacting with His57 and Ser195 (sticks). KLK4-PABA has two Ni^2+^ ions bound that are not seen in KLK4-Ni, on both sides of loop I. One Ni^2+^ ion is common between both structures, bound to His25 and Glu77. The KLK4-Ni structure is flexible and has missing electron density in loop III as depicted in the circled region; (**C**) Overlay of KLK4-Ni (solid) and KLK4-PABA (translucent). Loops II, IV, and VI–VIII are well aligned; (**D**) Overlay focused on loop III, showing missing electron density. Ni^2+^ was observed at this site in both structures.

Structural and mutagenesis data showed that KLK4 can be inhibited by a Ni^2+^ ion binding to residues more than 20 Å from the active site^23^. The KLK4-PABA structure contained 3 nickel ions bound in three separate remote sites (Fig. 5A). Mutagenesis experiments showed that mutation of the nickel-chelating residues His25 or Glu77 in one of the sites completely abolished metal mediated inhibition^23^. To gain more insight into this mode of inhibition, we crystallized KLK4 under identical conditions but in the absence of PABA inhibitor. The resulting 2.3 Å resolution structure (KLK4-Ni), a dimer in the asymmetric unit, contains a nickel ion bound to the His25/Glu77 site, but not to either of the previously reported loop I sites (Fig. 5B–D). We also show for the first time that in the absence of PABA, a nickel ion is bound to KLK4 catalytic residues His57 and Ser195, stabilized in the crystal by interactions with Gln183 of two symmetry-related molecules. It is therefore unlikely that this nickel site is physiologically relevant. All four nickel positions were validated by an anomalous difference electron density map calculation.

**Figure 6:**
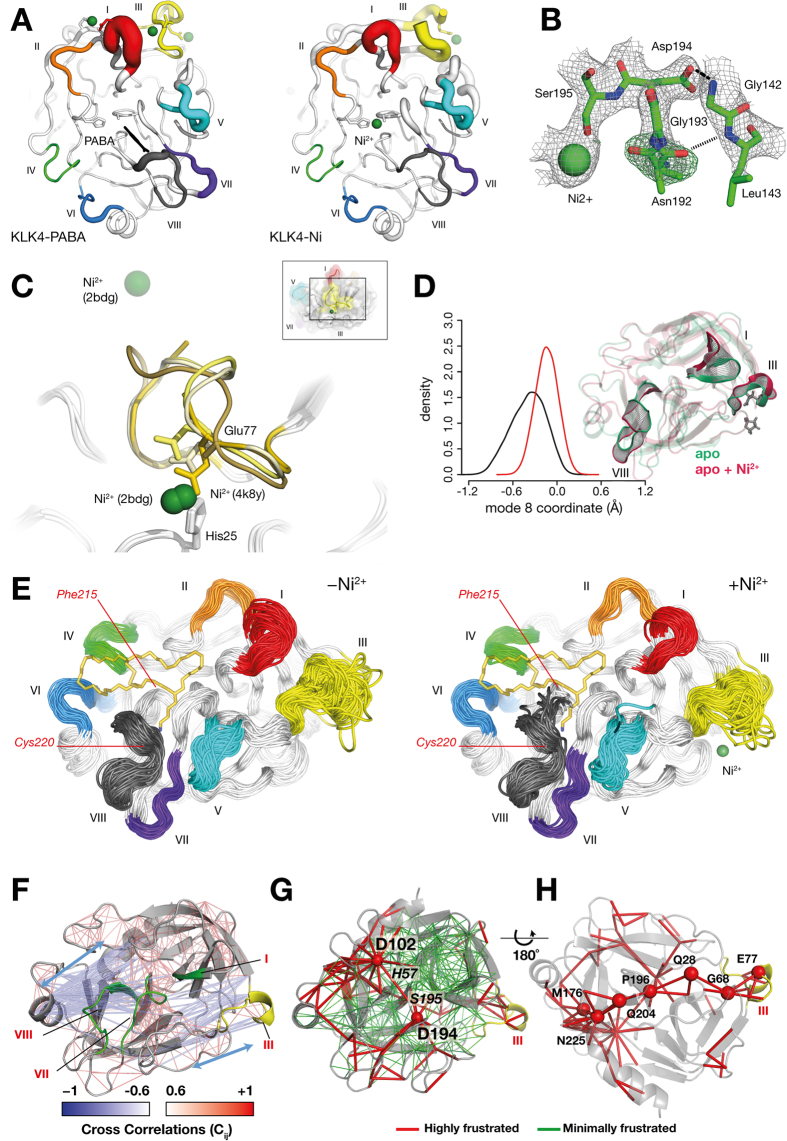
KLK4 Dynamics. (**A**) Normalised B-factor cartoons of KLK4-PABA (2BDG) and KLK4-Ni (4KGA), showing chain A alone. B-factors have been normalised within each individual structure. KLK4-PABA has an average B-factor of 20.59, and KLK4-Ni chain A has an average B-factor of 65.77; (**B**) Two conformations of the N192-G193 peptide bond in the KLK4-Ni crystal structure. Composite omit map indicates the presence of both conformations. 2*F*_o_ − *F*_c_ maps (grey) are shown at 1σ, composite omit *F*_o_ − *F*_c_ maps (green) are shown at 3σ. A H-bond is shown as long black dashes between D194^OD^-G142^N^, a H-bond only present in one conformation between N192^o^-L143^N^ is shown in short black dashes; (**C**) Loop III is structurally diverse. The structures of KLK4-Ni (golden), KLK4-SFTI-1_FCQR_ (brass), KLK4-SFTI (bright yellow) and KLK4-PABA (cream) are shown with nickel binding site residues H25 and E77 represented as sticks; (**D**) Nickel binding shifts the KLK4 equilibrium populations related to large amplitude motions. (*Left*) Normal mode coordinates were computed for each conformational state explored during the MD trajectories of KLK4-apo (black) and KLK4-Ni (red); (*Right*) cartoon representation of the most populated states showing that despite the distance from the Ni binding site (highlighted by its coordinating residues His25 and Glu77 in sticks), regions close to the active site (dashed circle) undergo noticeable conformational rearrangements; (**E**) Superposition of MD snapshots for KLK4 with and without Ni at the His25/Glu77 site. Snapshots at 1 ns intervals from the MD simulation of KLK4 without Ni (KLK4-apo, left) and with Ni (KLK4-Ni, right) are presented, SFTI is shown in the active site for context, but was not present in simulations; (**F**) Dynamical cross correlations (*C*_(*i*,*j*)_) calculated from motions contributed by the first 40 low frequency normal modes, plotted onto the KLK4-Ni structure. Red and blue edges connect pairs of residues that are correlated or anticorrelated, respectively; (**G**) Visualization of the frustration networks mapped onto the KLK4 structure. Minimally and highly frustrated contacts are represented in green and red, respectively; (**H**) Visualization of the intramolecular path of highly frustrated residues, connecting loop III (in yellow) to the active site region.

Inspection of B-factors together with discontinuous regions of electron density in loop III suggests that the KLK4-Ni structure is more flexible than the KLK4-PABA structure (Fig. 6A). Loop III flexibility is also consistent with missing electron density for loop III residues 74–75 and 73–74 in chains A and B respectively, even in the presence of stabilizing crystal contacts. Thus, as previously suggested^23^, without the stabilizing influence of the PABA active site inhibitor, the increased flexibility of the KLK4-Ni structure enables it to sample new conformations.

It has been shown that binding of a metal to the His25/Glu77 site alters the equilibrium of Ile16 insertion, and that this site is responsible for the metal-mediated inhibition of KLK4, since H25A or E77A mutants are no longer inhibited by metals^23^. With nickel bound to this site, we observe discrete structural heterogeneity in the active site of the KLK4-Ni structure; the Asn192-Gly193 peptide bond can be modelled in two conformations: a canonical conformation where the carbonyl of Asn192 forms a H-bond with the backbone of Leu143, and a non-standard, flipped Asn192 peptide bond with a loss of this H-bond (Fig. 6B). This non-standard orientation has also been noted in other serine protease structures^32^,^33^,^34^,^35^,^36^,^37^ and is chemically inhibitory since it negates the stabilizing effect of the oxyanion hole on the tetrahedral intermediate during catalysis. In addition to direct effects on catalysis, we propose loss of the Asn192-Leu143 H-bond will increase the dynamics of Leu143, and destabilize its interaction with Ile16, shifting structural equilibrium towards Ile16 release.

The simultaneous finding of flexibility in loop III adjoining the His25/Glu77 inhibitory metal binding site and inhibitory conformations at the active site suggests a correlation between these sites. Furthermore, loop III shows the highest degree of structural diversity when comparing KLK4-PABA, KLK4-Ni, KLK4-SFTI-1 and KLK4-SFTI-1_FCQR_ structures (Fig. 6C). Since the presence of active site ligands is known to alter the dynamics of serine proteases^38^,^39^ we suggest H25/E77-loop III and the active site are hubs of information transfer that regulate KLK4 activity depending upon ligand state.

### MD simulations reveal widespread changes in dynamics upon metal binding, hindering substrate access to the active site

Intrigued by how the His25/Glu77 metal binding site more than 20 Å from the active site indirectly inhibits proteolytic activity, we performed MD simulations of KLK4 in the presence and absence of nickel bound at this site. In the His25/Glu77 nickel-free KLK4 (KLK4-apo) simulation, loop III exhibits the most flexibility, compared to the rest of the molecule (Fig. 6E). Thus, MD simulations are consistent with conformational flexibility in loop III, as indicated by crystal structural analysis of KLK4-Ni, as well as the structural diversity amongst KLK4 structures. In simulations of the His25/Glu77 Ni-bound structure, despite preserving overall flexibility in loop III, the Glu77-Ni-His25 interaction is associated with a reduction in mobility (Fig. S3B). We also observed a long-range stabilization effect on distant regions such as active site loop II and the C-terminal half of loop VIII (Fig. 6E & S3B), associated with Ni^2+^ binding. In contrast, simulations of the Ni-bound structure show increased fluctuations around subsite S3, between Phe215 and Cys220 of loop VIII (Fig. 6E). Our simulations therefore suggest that metal binding to the His25/Glu77 site results in local stabilization (loop III) that affects overall KLK4 dynamics (loops II and VIII).

By assuming SFTI-1 residues P4-P2′ bind to KLK4 in a similar mode to a natural substrate, we can use the KLK4-SFTI structure to model the effect that MD-simulated loop motions would have on substrate binding. Superimposing the SFTI-1 structure onto the MD-generated ensemble of KLK4-apo simulations shows that the His25 and loop II, III and VIII motions do not interfere with SFTI-1 P4-P2′ binding and would not be inhibitory (Fig. 6E). Thus this simulation mostly sampled the non-inhibitory apo state (Fig. 6E). In contrast, SFTI superposition onto the Ni-bound ensemble reveals significant clashes at active site loop VIII (Fig. 6E). During the simulation timecourse, initially the Phe215 sidechain clashes with the P2/P3 residues of SFTI-1, followed by the 215–217 segment moving to clash with P1/P2, ending with a flip of Lys217 sidechain to clash with P2/P3/P4. Therefore, these simulations show how loop VIII motions would sterically hinder substrate binding when Ni^2+^ is bound to the His25/Glu77 site, offering mechanistic insight into metal-mediated indirect inhibition of proteolytic activity.

### Metal-dependent coupled loop dynamics drives long-range communication networks in KLK4

In order to explore the effect of metal-binding on large amplitude motions of KLK4 we calculated normal mode (NM) coordinates for each conformational state explored during the MD trajectories of the KLK4 in the presence and absence of nickel. Conformations explored during atomistic MD were projected onto the first five low-frequency NMs. With one key exception, the distribution of conformations was centered on the origin, showing limited sampling of rigid-body motions during MD (Fig. 6D & S4). However, the presence of nickel bound near loop III resulted in a shift in the equilibrium population along mode 8. This global motion links loop III (close to the Ni-binding site) with the loops I and VIII near the active site, indicating that Ni-binding affects the equilibrium conformation of KLK4. Next, dynamical cross correlation (DCC) coefficients were calculated for pairs of residues in KLK4 and mapped onto the structure (Fig. 6F). Importantly, loop III motions (near the allosteric site) were observed to be linked to those of loop VII residues and active site residues (especially Asp194), highlighting dynamic communication between these distant regions. We then investigated the distribution of energy within the KLK4 structure using a mutational frustration analysis, which explores the influence of localized sequence and conformational perturbations on the energetic frustrations^40^ within the structure. Most of the structure shows favorable interactions between residues (Fig. 6G), though both the active site region and loop III contain patches of highly frustrated contacts. These distant regions are connected by an intramolecular path of frustrated interactions (Fig. 6H), spanning from Glu77 (at loop III) to hubs of localized frustration at Asp102 and Asp194 in the active site (Fig. 6G). Taken together, this analysis indicates a correspondence between conformational dynamics and energetic frustrations on KLK4, and identifies a network of frustrated residues that may form a communication network bridging the Ni-binding site and the active site.

### SFTI-1 binding is correlated with reduced dynamics and frustration in loops VI and III

Loop VI, which is highly frustrated in the apo form, becomes more stabilized in the presence of SFTI-1_FCQR_, the more potent inhibitor (Fig. S5). The KLK4-SFTI-1_FCQR_ structure shows an alternative loop III conformation to those obtained in the KLK4-Ni and KLK4-SFTI-1 structures (Fig. S8A). This single, less-frustrated conformation (Fig. S8C) was maintained throughout all simulations of KLK4 with SFTI-1_FCQR_ (Fig. S8B). All other simulations with bound inhibitor showed at least two highly-populated states − similar to the distribution for the apo form − showing that these bound inhibitors poorly restrict the intrinsic conformational variability in loop III (Fig. S8C). This analysis suggests a dynamic interplay between the active site and loop III, supporting the observations that active site occupancy is associated with alternate loop III conformations in the crystal structures, and loop III instability is associated with non-canonical “inhibitory” conformations when the active site is empty.

### KLK4 and factor VIIa share a common metal-binding site

Kallikreins are proteases that perform catalysis in organs with high zinc concentrations^21^. The KLK4 inactivation by nickel described in this work is also proposed to apply to zinc binding to the same H25/E77 site^23^. A molecular understanding of zinc-mediated inhibition of members of the kallikrein family is emerging with three types of inhibition seen^41^. First, in a KLK3 structure, zinc has been shown to bind Asp91, His101 and His233. This is proposed to inhibit activity by changing the conformation of catalytic residue Asp102^42^. Second, zinc binding to residues in loop IV (also known as the 99-loop) and the catalytic His57 is proposed to block catalysis for rKLKL1c2^43^, KLK5^44^ and KLK7^45^. KLK4 is unique among the kallikreins in its mode of zinc-mediated inhibition through metal binding residues His25 and Glu77 in loop III. However, it has been noted previously that there are similarities between KLK4 and Factor VIIa with regards to allosteric inhibition by zinc^21^. FVIIa, when not bound to tissue factor, is inhibited by zinc but can reverse this inhibition with calcium^46^,^47^. A structural comparison between FVIIa^33^ and KLK4 reveals the structural equivalence of one (“site-2”) of the two zinc sites of FVIIa with the Ni/Zn His25/Glu77 site of KLK4 (Fig. S9). Therefore, both proteases feature a metal bridge between the N-terminal strand (at Lys24) and loop III. Since FVIIa also has a calcium ion that is chelated by loop III, we speculate that the calcium binding site in loop III is required for stability — without calcium, loop III movements could send site-2-dependent inhibitory signals to the active site, while calcium binding would stabilize loop III and reverse this inhibition. Thus we propose that KLK4 and FVIIa share similar mobile loop III/metal bridge allosteric inhibitory mechanisms.

## Conclusions

KLK4 has been crystallized in the presence of SFTI-1 and an engineered SFTI-1 derivative. These structures allow detailed insights into the interactions that occur between the active site and inhibitors, enabling more specific inhibitors to be designed for KLK4. Furthermore, our KLK4-Ni structure shows an inhibitory conformation for the 192–193 peptide bond, an active site-bound metal and implies loop III has instability/flexibility that has not been previously reported^23^. We propose that loop III flexibility is a key component in the indirect regulation of KLK4. This hypothesis is interesting from an evolutionary perspective. During evolution from ancestral trypsin^48^, KLK4 sacrificed some stability by mutation of an existing calcium-binding loop III while also acquiring a bridge for transferring instability in the form of an His25/Glu77 metal binding site. These molecular features would be favoured in the zinc- and calcium-rich environments of developing teeth^2^ in order to regulate KLK4 activity. Therefore, KLK4 divergence from ancestral trypsin is an adaptation to the specialized environments involved in development. Collectively, these studies offer insight into the structure and function of inhibition for the serine protease superfamily.

## Materials and Methods

### Protein expression, refolding and purification

Using the previously reported pET12-proPSA-hK4 chimera plasmid^49^, KLK4 was expressed in *E*.* coli* as inclusion bodies. The subsequent purification and refolding methods are described in detail in SI Methods.

### Synthesis of SFTI-1 variants

Inhibitory peptides were synthesized on 2-chlorotrityl resin (1.55 mmol/g, Iris Biotech) with 9-fluorenylmethyl carbamate as semi-permanent protecting group using a Discover SPS Microwave System (CEM Corporation) to enhance conventional solid phase peptide synthesis. Peptide cyclisation was carried out in solution also using microwave enhancement as previously described^17^.

### Inhibition assays

Inhibition of KLK4 by SFTI-1 was assessed in competitive inhibition assays, and the inhibition constant (K_i_) was determined by non-linear regression in GraphPad Prism (Morrison equation), as recently described^17^. Assays were performed three times in triplicate in 96-well low-binding plates (Corning) using 1.5 nM KLK4 and 120 μM FVQR-pNA in 250 μL assay buffer (0.1 M Tris-HCl pH 7.4, 0.1 M NaCl, 0.005% Triton X-100).

### Crystallization

All crystals were grown using the hanging drop vapor diffusion method, with 1:1 (v/v) ratio of protein to mother liquor.

#### KLK4-Ni.

Crystallization conditions for KLK4 in complex with *p*-aminobenzamidine (PABA)^23^ were used as a guide for crystallizing KLK4-Ni in absence of PABA. A gradient of 10–30% of PEG 2000 and Tris-HCl pH 7.5–9.0 was used for initial screening with KLK4 concentration at 10 mg/mL. Final crystals were grown at 18 °C using 4 μL drops equilibrated over 1 mL of 20% PEG 2000, 100 mM Tris pH 8.0, 10 mM NiCl_2_ buffer.

#### KLK4-SFTI-1 and KLK4-SFTI-1_FCQR_.

 Prior to crystallization experiments of the KLK4-SFTI-1 and KLK4-SFTI-1_FCQR_ complexes, 20 mg/ml protein was incubated overnight with 3-fold molar excess of inhibitor (solubilized in 25% acetonitrile to the working concentration of 100 mM) at 4 °C. In order to identify initial hits, commercially available screens including Index (Hampton Research), JCSG+ and PACT (Qiagen) were used^50^. The crystallization trials were set up manually at 18 °C utilizing hanging drop vapor diffusion method. Final KLK4-SFTI-1_FCQR_ crystals were obtained within a week by mixing 2 μL of protein-peptide solution with equal volume of crystallization buffer (0.1 M Li_2_SO_4_, 0.1 M sodium acetate, 30% PEG 8000, pH 4.6) and equilibrated at 18 °C. KLK4-SFTI-1 crystals were produced by mixing 2 μL of protein-peptide solution and 2 μL of reservoir buffer (0.2 M Li_2_SO_4_, 0.1 M sodium acetate, 20% PEG 8000, pH 4.8) and appeared after 9 months of incubation at 18 °C.

### X-ray data collection, structure determination and refinement

All datasets were collected at the Australian Synchrotron, Victoria, Australia on MX1^51^ (KLK4-Ni) and MX2 (KLK4-SFTI complexes) beamlines using the Blu-Ice software^52^. More detail can be found in SI Methods. Structure determination proceeded using the molecular replacement method and the program PHASER^53^. A search model was constructed from the crystal structure of KLK4 in complex with PABA^23^ (PDB: 2BDG, chain A) by removing solvent molecules and ligands. All subsequent model building, refinement and structural validation was done using Phenix^54^ and COOT^55^. Composite omit maps (Fig. 6C) were calculated using Phenix^54^. An anomalous difference map was created for the KLK4-Ni structure (using anomalous difference structure factor amplitudes and phases generated from the refined structure with heavy atoms removed). Four peaks were observed on this map at between 4.3 to 4.8 σ, supporting the positions of the Ni atoms. A summary of statistics is provided in Table 1.

### Structure analysis

For all analysis and MD simulations, missing atoms, side chains and residues were rebuilt using Modeller v9.10^56^. In each instance, 50 models were built and the lowest DOPE (Discrete Optimized Protein Energy) scoring model was selected for further analysis. Hydrogen bonding and salt bridge values were calculated using the PISA web-server^57^. Solvent accessible surface area was calculated using AREAIMOL as part of the ccp4 package with a default probe radius of 1.4 Å^58^. Structural comparisons between KLK4, SFTI-1 and related serine proteases discussed in the text were performed after a global backbone alignment using the following PDB entries: SFTI-1 NMR structure (1JBL), KLK4-PABA (2BDG), trypsin-SFTI-1 (1SFI), trypsin-benzamidine (2BLV), matriptase-SFTI-1 (3P8F), matriptase-benzamidine (1EAX) and ligand-free matriptase (4IS5). Comparisons to determine structural changes induced/selected by SFTI-1 binding were performed by inspection of structural deviations between SFTI-1 bound and corresponding benzamidine/PABA bound proteases structures. When 3 consecutive residues or more were found to have more than 0.5 Å Cα deviation, this deviation was then compared against a third structure with an unliganded active site. If the deviation was only seen in the SFTI-1 structure (determined statistically by comparing values in a two-tailed T-test), the structural change was marked as being induced/selected by SFTI-1.

### Computational resources

Calculations, modeling and simulations were performed on a range of computing resources: ORCHARD 800 core x86 cluster (Monash University; X-ray ensemble refinement); AVOCA/MERRI (VLSCI BlueGene/Q/x86 cluster; atomistic MD).

### Atomic coordinates, modeling and graphics

In MD simulations, atomic coordinates were obtained from the following PDB entries: 4KGA (chain A), 4K8Y & 4K1E. Missing residues and atoms were rebuilt using MODELLER version 9.10^56^. All structural representations were produced using PyMOL version 1.7.6^59^ and VMD 1.9.2^60^, and all trajectory manipulation and analysis was performed with a combination of custom scripts, MDTraj^61^, SciPy^62^, Matplotlib^63^, iPython^64^ and VMD 1.9.2^60^.

### Molecular dynamics (MD) systems setup and simulation

Each protein, with protonation states appropriate for pH 7.0^65^,^66^, was placed in a rectangular box with a border of at least 12 Å, explicitly solvated with TIP3P water^67^, counter-ions added, and parameterized using the AMBER ff14SB all-atom force field^68^,^69^,^70^. Harmonic restraints were added to maintain the Ni^2+^ ion bound at the His25 and Glu77 site.

After an energy minimization stage, and an equilibration stage, production simulations were performed in the NPT ensemble. Three independent replicates of each system were simulated for 200 ns each using NAMD 2.9^71^. More details are available in SI Methods.

### Normal mode calculations

The normal modes of KLK4-apo were calculated with CHARMM 37^72^ software in conjunction with the AMBER ff99SB forcefield^73^. Calculations were performed in vacuum using a distance dependent dielectric constant (*ε* = *2r*_*i*,*j*_), to treat electrostatic interactions. Prior to NM calculations, the KLK4-apo structure was energy minimized using the steepest descent (SD) and conjugate-gradient (CG) methods followed by the Adopted Basis Newton-Raphson (ABNR) algorithm. The energy minimized structure presented 0.7 Å RMSD (backbone atoms) against the crystallographic conformation. Harmonic restraints were applied during the SD steps and were progressively decreased from 250 to 0 kcal mol^−1^ Å^−2^. Then, the system was further energy minimized with 1000 CG steps and the ABNR algorithm applied without positional restraints using a convergence criterion of 10^−5 ^kcal mol^−1^ Å^−1^ RMS energy gradient. The first 100 low frequency normal modes and the atomic fluctuations were computed with the VIBRAN module of CHARMM. The first five low frequency NMs (Movie S1) accounted for 68% of overall KLK4 dynamics.

### Dynamical cross correlation analysis

Rotational and translational degrees of freedom were removed by global backbone alignment. Following this, dynamical cross correlation coefficients between pairs of residues were calculated considering a set of 100 NMs using the R program with the bio3D package^74^.

### Local Frustration Analysis

Local frustration analysis was conducted with the Frustratometer web server^75^. Briefly, the energetic frustration is obtained by the comparison of the native state interactions to a set of generated “decoy” states where the identities of each residue are mutated. Each contact is defined as “minimally frustrated” or “highly frustrated” upon comparison of its frustration energy with values obtained from the decoy states as described^40^.

## Additional Information

**Accession Codes:** The coordinates and structure factors have been deposited in the Protein Data Bank under accession codes 4KGA (KLK4-Ni), 4K8Y (KLK4-SFTI-1) and 4K1E (KLK4-SFTI-1_FCQR_).

**How to cite this article**: Riley, B. T. *et al*. Direct and indirect mechanisms of KLK4 inhibition revealed by structure and dynamics. *Sci. Rep.* **6**, 35385; doi: 10.1038/srep35385 (2016).

## Supplementary Material

Supplementary Information

Supplementary Movie S1

Supplementary Movie S2

## Figures and Tables

**Table 1:** Data collection and refinement statistics^1^.

*Data collection*	KLK4-SFTI-1	KLK4-SFTI-1_FCQR_	KLK4-Ni
Wavelength (Å)	0.8266	0.9537	0.9537
Space group	*P*12_1_1	*P*12_1_1	*P*4
Unit cell dimensions a, b, c (Å), α, β, γ (°)	31.93, 75.75, 39.74 90, 95.73, 90	39.78, 63.31, 41.1890, 115.66, 90	99.31, 99.31, 41.0290, 90, 90
Resolution (Å)	29.3-1.0	37.1-1.30	70.22-2.32
Number of measured reflections	400653	159204	123253
Number of unique reflections	100687	45068	17693
Completeness (%)	99.5 (99.6)	99.6 (100.0)	100.0 (100.0)
Redundancy	4.0 (3.9)	3.5 (3.4)	7.0 (5.8)
*R*_pim_	0.054 (0.253)	0.053 (0.188)	0.039 (0.359)
CC1/2	0.998 (0.869)	0.990 (0.807)	0.475 (0.280)
<*I*/σ*I*>	8.7 (2.7)	9.6 (4.2)	12.9 (2.1)
*Structure refinement*			
Number of reflections	100641	45043	17689
Number of protein atoms	1924	1798	3214
Number of water molecules	326	115	56
* R*_*work*_ (%)	15.0	14.0	22.0
* R*_*free*_ (5% of data) (%)	17.0	17.0	26.4
RMSD bond lengths (Å)	0.006	0.008	0.003
RMSD bond angles (°)	1.21	1.16	0.91
Average B-factor (Å^2^)			
Protein	10.121	12.306	66.026
Inhibitor	13.328	18.399	—
Solvent	21.848	19.456	54.708
Ramachandran			
Favoured (%)	98.82	97.53	97.95
Outliers (%)	0	0	0
MolProbity score	0.86, 99th percentile (N = 666, 1.00 Å ± 0.25 Å)	0.79, 100^th^ percentile (N = 2276, 1.30 Å ± 0.25 Å)	1.37, 100^th^ percentile (N = 8665, 2.32 Å ± 0.25 Å)
PDB ID	4K8Y	4K1E	4KGA

^1^Values in parentheses are for high resolution shell.
