# Supplementary material for: Direct and indirect mechanisms of KLK4 inhibition revealed by structure and dynamics
Source: Sci Rep. 2016 Oct 21;6:35385. doi: 10.1038/srep35385 (PMC5073354; doi:10.1038/srep35385)
Supplement: Supplementary Information [file srep35385-s1.pdf]

Supporting information:

Direct and indirect mechanisms of KLK4 inhibition revealed by structure and dynamics

Blake T. Riley, Olga Ilyichova, Mauricio G.S. Costa, Benjamin T. Porebski, Simon J. de Veer, Joakim E. Swedberg, Itamar Kass, Jonathan M. Harris, David E. Hoke, and Ashley M. Buckle

SI Methods

Protein expression, refolding and purification

The expression of KLK4 was carried out in *E.coli* using previously reported pET12-proPSA-hK4 chimera plasmid[1]. In this expression vector KLK4 pro-region was replaced with the seven amino acid pro-region of PSA to enable auto activation. The protease was expressed as inclusion bodies that were isolated and solubilised according to the previously published methods[1,2] with some modifications. KLK4 activity was tested by its ability to form SDS-resistant complexes with alpha-1 antitrypsin (data not shown).

The expression plasmid was heat-shock transformed into *E. coli* BL21(DE3) pLysS cells and grown at 37°C in 2 litres of 2xLB medium containing 100 μg/ml ampicillin and 34 μg/ml chloramphenicol until the absorbance A_{600} reached 1.0. The expression of KLK4 was induced with 1 mM isopropyl-β-thiogalactoside and incubation continued for an additional 2-4 hours. The cells were harvested by centrifugation at 10000g for 20 min at 4°C. The resultant pellet was resuspended in 20 ml of lysis buffer (25% sucrose, 50 mM Tris-HCl, 2 mg/mL lysozyme, 5 mM $MgCl_2$, pH 8.0) and incubated at 4°C until viscous, followed by sonication. The extract was treated with 10 μg/ml of DNAse I for 30 min at room temperature. The inclusion bodies were collected by centrifugation at 20000g for 1 hour at 4°C.

The pellets were then washed with 100 ml of detergent buffer (200 mM NaCl, 1% IGEPAL, 5 mM EDTA, 20 mM Tris-HCl, pH 7.5), and centrifuged at 10000g for 10 min at 4°C . Finally, the

inclusion body pellets were washed with 100 mL of 200 mM NaCl, 20 mM Tris-HCl, 1 mM EDTA, 0.5% Triton X-100, pH 7.5, and centrifuged at 10000g for 10 min at 4°C. The last step was repeated three times.

90% pure KLK4 was solubilised in 20 mL of 8M Urea, 150 mM NaCl, 100 mM NH_4Cl, 100 mM β-mercaptoethanol, 2 mM EDTA, 50 mM Tris-HCl, pH 8.5. The solubilised protein was then refolded by diluting into 2 L of 0.5M Urea, 100 mM NaCl, 7 mM GSH, 0.7 mM GSSG, 2 mM $CaCl_2$, 0.5 M L-Arginine, 50 mM Tris-HCl, pH 8.0 for 16 hours at 4°C. The sample was concentrated to 20 ml by ultrafiltration using VF20P0 Vivaflow 200 membrane (Sartorius Stedim) and dialyzed against 5 L of 50 mM Tris-HCl pH 7.5, 100 mM NaCl, 2 mM $CaCl_2$ overnight at 4°C. The sample was then applied to a pre-equilibrated (50mM Tris-HCl pH 7.5, 20 mM NaCl, 2 mM $CaCl_2$) HiLoad 16/60 Superdex 75 prep grade gel filtration column and eluted with the same buffer at 1.0 ml/min. Active KLK4 was found in the second peak.

X-ray data collection, structure determination and refinement

All datasets were collected at the Australian Synchrotron, Victoria, Australia on MX1 (KLK4-Ni) and MX2 (KLK4-SFTI complexes) beamlines using the Blu-Ice software[3]. MX1 beamline was equipped with an ADSC Quantum 210r detector and MX2 with an ADSC Quantum 315r Detector respectively[4]. For cryoprotection the crystals were transferred into a solution of mother liquor with 20% (v/v) MPD and flash-cooled in liquid nitrogen. All datasets were integrated, merged and scaled with iMosflm and Scala[5] from the CCP4 suite[6,7]. Each dataset was processed in *P*1 and Laue group determination was achieved using Pointless. Datasets were scaled and merged in their respective space-group and 5% of each dataset was flagged for calculation of R_{free}, with neither a sigma nor a low-resolution cut-off applied to any dataset. A summary of statistics is provided in Table 1. Structure determination proceeded using the molecular replacement method and the program PHASER[8]. A search model was constructed from the crystal structure of KLK4 in complex with PABA[9] (PDB: 2BDG, chain A) by removing solvent molecules and ligands. All subsequent model

building, refinement and structural validation was done using Phenix[10] and COOT[11]. Composite omit maps (Fig. 6C) were calculated using Phenix[10]. An anomalous difference map was created for the KLK4-Ni structure (using anomalous difference structure factor amplitudes and phases generated from the refined structure with heavy atoms removed). Four peaks were observed, with heights between 4.3 to 4.8 σ, supporting the positions of the Ni atoms.

Molecular dynamics (MD) systems setup and simulation

Residue protonation states were set as appropriate at pH 7.0 using PROPKA[12,13]. Each protein was then placed in a rectangular box with a border of at least 12 Å of water on all sides of the protein, and the system charge was neutralized by addition of sodium or chloride counter-ions. Systems were parameterized using the AMBER ff14SB all-atom force field[14-16] in conjunction with the TIP3P explicit water model[17]. To maintain the Ni^{2+} ion bound at the His25 and Glu77 site, distances were restrained with a force constant of 100 $kcal^{-1}$ mol^{-1} $Å^{-2}$ using the distances measured in the crystal structure as a guide.

Systems were relaxed with 15000 steps of energy minimization, followed by equilibration. In equilibration, atoms' initial velocities were randomly distributed according to a Maxwell-Boltzmann distribution at 100 K. Harmonic positional restraints of 100 $kcal^{-1}$ mol^{-1} $Å^{-2}$ were applied to protein backbone atoms and temperature was steadily increased from 100 K to 300 K over the course of 100 ps, with a Langevin damping coefficient of 5 ps^{-1}. Pressure was then equilibrated to 1 atm with a Berendsen barostat[18] (τ_p = 0.1 ps) and restraints were removed steadily over 200 ps.

Production simulations were performed in the NPT ensemble without positional restraints, using an integration timestep of 2 fs, and saving snapshots every 5 ps for analysis. Three independent replicates of each system were simulated for 200 ns each. All simulations were performed using NAMD 2.9[19] with periodic boundary conditions, long-range interactions were computed using PME[20] with an 8 Å cutoff radius.

Normal mode calculations

The normal modes of KLK4-apo were calculated with CHARMM 37[21] software in conjunction with the AMBER ff99SB forcefield[22]. Calculations were performed in vacuum using a distance dependent dielectric constant ($\varepsilon=2r_{i,j}$), to treat electrostatic interactions. Prior to NM calculations, the KLK4-apo structure was energy minimized using the steepest descent (SD) and conjugate-gradient (CG) methods followed by the Adopted Basis Newton-Raphson (ABNR) algorithm. The energy minimized structure presented 0.7 Å RMSD (backbone atoms) against the crystallographic conformation. Harmonic restraints were applied during the SD steps and were progressively decreased from 250 to 0 kcal mol^{-1} Å^{-2}. Then, the system was further energy minimized with 1000 CG steps and the ABNR algorithm applied without positional restraints using a convergence criterion of 10^{-5} kcal mol^{-1} Å^{-1} RMS energy gradient. The first 100 low frequency normal modes and the atomic fluctuations were computed with the VIBRAN module of CHARMM. The first five low frequency NMs (Movie S1) accounted for 68 % of overall KLK4 dynamics.

SI Tables, Figures and Movies

Table S1. KLK4 residues that interact closely with SFTI-1 (residues with atoms within 4Å radius) and the KLK4 residues involved in H-bonds and salt bridges with SFTI-1 (as calculated by PISA[23]). KLK4 residue loop designations are also indicated. Cells with solid lines (____) indicate a lack of KLK4 residues that are within 4Å / interact by H-bonds or salt bridges. Where SFTI-1 residues in the crystal structure could not be modelled, interaction data is not applicable (N/A) as denoted.

**SFTI-1 res# / aa / specificity**	**KLK4 residues <4Å**	**KLK4 loops**	**H-bonds: SFTI-KLK4**	**Salt bridges: SFTI-KLK4**
1/G/P5	216GKA	VIII	Gly^{O}1-Ala^{N} 218	____
2/R/P4	L171 / 216GK	VI / VIII	Arg^{NH2}2-Leu^{O}171	____
3/C/P3	215FG	VIII	Cys^{N}3-Gly^{O}216<br>Cys^{O}3-Gly^{N}216	____
4/T/P2	H57 / N192 / 214SF	II / VII / VIII	____	____
5/K/P1	189DSCNGDS / 213VSF	VII / VIII	Lys ^{N}5-Ser ^{O}214<br>Lys ^{NZ}5-Ser ^{OG}190<br>Lys ^{O}5-Ser ^{N}195<br>Lys ^{O}5-Gly ^{N}193	Lys^{NZ}5-Asp^{OD1}189<br>Lys^{NZ}5-Asp^{OD2}189
6/S/P1'	41FC / H57 / 192NG / S195	I / II / VII / VII	Ser ^{N}6-Ser ^{OG}195	____
7/I/P2'	40LF / M149 / 192NG	I / V / VII	Ile ^{N}7-Phe ^{O}41	____
8/P	____	____	____	____
9/P	N192	VII	____	____
10/I	H57	II	____	____
11/C	____		____	____
12/F	N95/L99	IV	____	____
13/P	N/A	N/A	N/A	N/A
14/D	N/A	N/A	N/A	N/A

Table S2. Comparison of H-bonding / salt-bridge interactions and buried surface area in SFTI-1 complexes with KLK4, trypsin and matriptase. Backbone and sidechain H-bonds are indicated by BB H-, and SC H-, respectively. Cells with solid lines (____) indicate a lack of KLK4 residues that interact by H-bonds or salt bridges. Where SFTI-1 residues in the crystal structure could not be modelled, interaction data is not applicable (N/A) as denoted.

	**KLK4-SFTI-1**		**Trypsin-SFTI-1**		**Matriptase-SFTI-1**	
**SFTI-1 residue**	**H-bonds/salt bridges**	**buried surface area Å^2**	**H-bonds/salt bridges**	**buried surface area Å^2**	**H-bonds/salt bridges**	**buried surface area Å^2**
G	1BB H-	28	1BB H-	24	1BB H-	35
R	1SC H-	130	1SC H-	120	3SC H-	138
C	2BB H-	41	2BB H-	42	2BB H-	55
T	____	54	1 BB H-	55	1BB H-	66
K	3BB H-<br>1SC H-<br>2SC salt	201	3BB H-<br>2SC H-<br>2SC salt	208	3BB H-<br>2SC H-<br>1SC salt	206
S	1BB H-	43	1BB H-	46	1BB H-	50
I	1BB H	114	1BB H-	140	1BB H-	117
P	____	0	____	6	____	39
P	____	28	____	17	____	14
I	____	28	____	29	____	53
C	____	0	____	0	____	1
F	____	90	____	57	____	23
P	N/A	N/A	____	0	____	0
D	N/A	N/A	____	19	____	22

Table S3. Interactions between SFTI-1_{FCQR} and KLK4. KLK4 residues within 4 Å of SFTI-1_{FCQR} residues, their loop designations, H-bonds and salt bridges.

**SFTI-1_{FCQR} res / aa / specificity**	**KLK4 residues <4 Å**	**KLK4 loops**	**H-bonds: FCQR - KLK4**	**salt bridges: FCQR - KLK4**
1/G/P5	216GKA	VIII	$Gly^{O}1$ - $Ala^{N}218$	____
2/F/P4	L99 / 172YD / 175L / 216GK	IV / VI / VIII	____	____
3/C/P3	215FG / 218A	VIII	$Cys^{N}3$ - $Gly^{O}216$<br>$Cys^{O}3$ - $Gly^{N}216$	____
4/Q/P2	H57 / Y94 / L99 / 214SF	II / IV / IV / VIII	$Gln^{NE2}4$ - $Tyr^{OH}94$	____
5/R/P1	H57 / 189DSCNGDS / 214SFGK / C220 / G226	II / VII / VIII / VIII / sheet C-term of VIII	$Arg^{N}5$ - $Ser^{O}214$<br>$Arg^{NH1}5$ -$Ser^{OG}190$<br>$Arg^{NH1}5$ - $Ser^{O}190$<br>$Arg^{NH2}5$ - $Lys^{O}217$<br>$Arg^{O}5$ - $Ser^{N}195$<br>$Arg^{O}5$ - $Gly^{N}193$	$Arg^{NH1}5$ - $Asp^{OD1}189$<br>$Arg^{NH1}5$ - $Asp^{OD2}189$<br>$Arg^{NH2}5$ - $Asp^{OD2}189$
6/S/P1'	41FC / H57 / 192NG / S195	I / II / VII / VII	$Ser^{N}6$ - $Ser^{OG}195$	____
7/I/P2'	40LF / M151 / 192NG	I / V / VII	$Ile7^{N}$ - $Phe41^{O}$	____
8/P	____	____	____	____
9/P	N192	VII	____	____
10/I	H57	II	____	____
11/C	____	____	____	____
12/F	L99	IV	____	____
13/P	____	____	____	____
14/D	L175	VI	____	____

Table S4. Polar interactions between symmetry mates of crystal structures. Symmetry partners are denoted by their symmetry operator (e.g. #2(-X,Y+½,Z)) followed by the required translation along lattice vectors. Interacting atoms are designated by chain, residue, atom and surface loop (if applicable).

Mol	Chn	Res	Atm	Loop	Mol	Chn	Res	Atm	Loop	Dist/ Å
4KGA	A	SER214	O		#2(-X,-Y, Z) + (0,-1, 0)	A	GLN186A	NE2	loop 7	2.816
4KGA	B	GLN76	N	loop 3	#1(X, Y, Z) + (0, 0,-1)	B	SER125	OG		3.473
4KGA	B	ASP75	N	loop 3	#1(X, Y, Z) + (0, 0,-1)	B	CYS232	O		3.406
4KGA	B	ASP75	N	loop 3	#1(X, Y, Z) + (0, 0,-1)	B	THR235	OG1		2.997
4KGA	B	GLN186A	NE2	loop 7	#2(-X,-Y, Z) + (0, 0, 0)	B	SER214	O		3.153
4KGA	B	GLN243	O		#3(-Y, X, Z) + (0, 0, 0)	A	ARG90	NH1		3.456
4KGA	B	GLN243	O		#3(-Y, X, Z) + (0, 0, 0)	A	ARG90	NH2		2.806
4KGA	A	ARG90	NH2		#3(-Y, X, Z) + (0, 0, 0)	B	GLN243	O		2.805
4KGA	A	ARG90	NH1		#3(-Y, X, Z) + (0, 0, 0)	B	GLN243	O		3.456
4KGA	B	SER214	O		#4(Y,-X, Z) + (0, 0, 0)	B	GLN186A	NE2	loop 7	3.153
4KGA	B	ASP102	OD2		#4(Y,-X, Z) + (0, 0, 0)	B	GLN186A	NE2	loop 7	3.577
4KGA	B	THR235	OG1		#1(X, Y, Z) + (0, 0, 1)	B	ASP75	N	loop 3	2.997
4KGA	B	CYS232	O		#1(X, Y, Z) + (0, 0, 1)	B	ASP75	N	loop 3	3.406
4KGA	B	SER125	OG		#1(X, Y, Z) + (0, 0, 1)	B	GLN76	N	loop 3	3.473
4KGA	A	GLN186A	NE2	loop 7	#4(Y,-X, Z) + (1, 0, 0)	A	SER214	O		2.816

Mol	Chn	Res	Atm	Loop	Mol	Chn	Res	Atm	Loop	Dist/ Å
#1(X,Y , Z) + (-1, 0, 0)	A	ARG90	NH1		4K8Y	A	ASN202	O		2.827
#1(X,Y , Z) + (-1, 0, 0)	A	ASN95	ND2	loop 4	4K8Y	A	ASN134	OD1		3.511
#1(X,Y , Z) + (-1, 0, 0)	A	ASN95	ND2	loop 4	4K8Y	A	ASN202	OD1		2.856
#1(X,Y , Z) + (-1, 0, 0)	A	ARG96	NH1	loop 4	4K8Y	A	THR130	OG1		3.213
#1(X,Y , Z) + (-1, 0, 0)	A	ARG96	NH1	loop 4	4K8Y	A	CYS128	O		2.901
#1(X,Y , Z) + (-1,	A	ARG96	NH	loop	4K8Y	A	CYS128	O		2.985

0, 0)			2	4						
#1(X,Y , Z) + (-1, 0, 0)	A	ARG96	NH2	loop 4	4K8Y	A	GLN127	OE1		3.163
4K8Y	A	SER135	OG		#1(X,Y , Z) + (-1, 0, 0)	B	CYS11	O		3.483
4K8Y	B	ARG2	NH2		#2(-X,Y+½,-Z) + (0,-1,-1)	A	GLU74	OE1	loop 3	2.853
4K8Y	A	LYS217	NZ	loop 8	#2(-X,Y+½,-Z) + (0,-1,-1)	A	GLU74	OE2	loop 3	3.269
4K8Y	B	ARG2	NH1		#2(-X,Y+½,-Z) + (0,-1,-1)	A	GLU74	OE2	loop 3	2.978
#2(-X,Y+½,-Z) + (0,-1,-1)	A	ARG150	NH2	loop 5	4K8Y	A	ASP173	OD1	loop 6	2.369
#2(-X,Y+½,-Z) + (0,-1,-1)	A	ARG150	NH2	loop 5	4K8Y	A	ASP173	OD2	loop 6	2.226
#2(-X,Y+½,-Z) + (0,-1,-1)	A	ARG150	NH2	loop 5	4K8Y	A	ASP173	OD1	loop 6	2.803
#2(-X,Y+½,-Z) + (0,-1, 0)	A	GLY79	N	loop 3	4K8Y	A	GLU93	OE2		2.853
#2(-X,Y+½,-Z) + (0,-1, 0)	A	GLY79	N	loop 3	4K8Y	A	GLU93	OE1		2.845
4K8Y	A	LYS233	NZ		#2(-X,Y+½,-Z) + (0,-1, 0)	A	GLU84	OE1		2.782
4K8Y	A	ARG150	NH1	loop 5	#1(X,Y , Z) + (0, 0,-1)	A	GLN50	OE1		2.909
4K8Y	A	ARG150	NH1	loop 5	#1(X,Y , Z) + (0, 0,-1)	A	SER111	OG		2.505
4K8Y	A	ASN192	ND2		#1(X,Y , Z) + (0, 0,-1)	A	GLN243	O		3.105
#1(X,Y , Z) + (0, 0,-1)	A	GLN243	NE2		4K8Y	A	CYS220	O	loop 8	3.274
4K8Y	A	CYS220	N	loop 8	#1(X,Y , Z) + (0, 0,-1)	A	GLN243	OE1		2.831
4K8Y	A	ARG150	NH2	loop 5	#2(-X,Y+½,-Z) + (0, 0,-1)	A	ASP173	OD1	loop 6	2.369
4K8Y	A	ARG150	NH2	loop 5	#2(-X,Y+½,-Z) + (0, 0,-1)	A	ASP173	OD1	loop 6	2.802
4K8Y	A	ARG150	NH2	loop 5	#2(-X,Y+½,-Z) + (0, 0,-1)	A	ASP173	OD2	loop 6	2.225
#2(-X,Y+½,-Z) + (0, 0,-1)	A	LYS217	NZ	loop 8	4K8Y	A	GLU74	OE2	loop 3	3.268
#2(-X,Y+½,-Z) + (0, 0,-1)	B	ARG2	NH1		4K8Y	A	GLU74	OE2	loop 3	2.979
#2(-X,Y+½,-Z) + (0, 0,-1)	B	ARG2	NH2		4K8Y	A	GLU74	OE1	loop 3	2.852
#2(-X,Y+½,-Z) + (0, 0,-1)	B	ARG2	NH1		#2(-X,Y+½,-Z) + (0, 0,-1)	B	ASP14	O		2.759
4K8Y	A	GLY79	N	loop 3	#2(-X,Y+½,-Z) + (0, 0, 0)	A	GLU93	OE1		2.845
4K8Y	A	GLY79	N	loop 3	#2(-X,Y+½,-Z) + (0, 0, 0)	A	GLU93	OE2		2.852
#2(-X,Y+½,-Z) + (0, 0, 0)	A	LYS233	NZ		4K8Y	A	GLU84	OE1		2.781

#1(X,Y , Z) + (0, 0, 1)	A	ARG150	NH1	loop 5	4K8Y	A	GLN50	OE1		2.908
#1(X,Y , Z) + (0, 0, 1)	A	ARG150	NH1	loop 5	4K8Y	A	SER111	OG		2.506
#1(X,Y , Z) + (0, 0, 1)	A	ASN192	ND2		4K8Y	A	GLN243	O		3.104
#1(X,Y , Z) + (0, 0, 1)	A	CYS220	N	loop 8	4K8Y	A	GLN243	OE1		2.832
4K8Y	A	GLN243	NE2		#1(X,Y , Z) + (0, 0, 1)	A	CYS220	O	loop 8	3.274
4K8Y	A	ARG96	NH2	loop 4	#1(X,Y , Z) + (1, 0, 0)	A	GLN127	OE1		3.162
4K8Y	A	ARG96	NH1	loop 4	#1(X,Y , Z) + (1, 0, 0)	A	CYS128	O		2.901
4K8Y	A	ARG96	NH2	loop 4	#1(X,Y , Z) + (1, 0, 0)	A	CYS128	O		2.986
4K8Y	A	ARG96	NH1	loop 4	#1(X,Y , Z) + (1, 0, 0)	A	THR130	OG1		3.214
4K8Y	A	ASN95	ND2	loop 4	#1(X,Y , Z) + (1, 0, 0)	A	ASN134	OD1		3.512
#1(X,Y , Z) + (1, 0, 0)	A	SER135	OG		4K8Y	B	CYS11	O		3.482
4K8Y	A	ARG90	NH1		#1(X,Y , Z) + (1, 0, 0)	A	ASN202	O		2.827
4K8Y	A	ASN95	ND2	loop 4	#1(X,Y , Z) + (1, 0, 0)	A	ASN202	OD1		2.856

Mol	Chn	Res	Atm	Loop	Mol	Chn	Res	Atm	Loop	Dist/Å
#2(-X,Y+½,-Z) + (-1,-1,-1)	A	SER88	N		4K1E	A	ASP116	OD2		2.767
#1(X,Y , Z) + (-1, 0,-1)	A	GLY133	N		#1(X,Y , Z) + (-1, 0,-1)	A	VAL162	O		2.913
#1(X,Y , Z) + (-1, 0,-1)	A	LYS170	NZ		4K1E	A	SER86	O		2.617
#1(X,Y , Z) + (-1, 0,-1)	A	LYS170	NZ		4K1E	A	ASN61	O	loop 2	2.641
#1(X,Y , Z) + (-1, 0,-1)	A	LYS170	NZ		4K1E	A	ASN61	OD1	loop 2	3.32
4K1E	A	SER88	N		#2(-X,Y+½,-Z) + (-1, 0,-1)	A	ASP116	OD2		2.767
#1(X,Y , Z) + (-1, 0, 0)	A	ARG150	NH1	loop 5	4K1E	A	SER111	OG		3.401
#1(X,Y , Z) + (-1, 0, 0)	A	ASN192	ND2		4K1E	A	GLN243	O		3.02
#1(X,Y , Z) + (-1, 0, 0)	A	CYS220	N	loop 8	4K1E	A	GLN243	OE1		2.839
4K1E	A	GLN243	NE2		#1(X,Y , Z) + (-1, 0, 0)	A	CYS220	O	loop 8	3.449
4K1E	A	SER135	OG		#2(-X,Y+½,-Z) + (0,-1, 0)	A	ASN95	O	loop 4	2.752
4K1E	A	SER23	OG		#2(-X,Y+½,-Z) + (0,-1, 0)	A	GLU165	OE2		3.01

4K1E	A	SER23	OG		#2(-X,Y+½,-Z) + (0,-1, 0)	A	GLU165	OE 2		2.478
#2(-X,Y+½,-Z) + (0,-1, 0)	A	SER169	OG		4K1E	A	HIS25	O		3.418
#2(-X,Y+½,-Z) + (0,-1, 0)	A	LYS170	NZ		4K1E	A	ASP116	OD 1		2.877
#2(-X,Y+½,-Z) + (0, 0, 0)	A	SER23	OG		4K1E	A	GLU165	OE 2		2.478
#2(-X,Y+½,-Z) + (0, 0, 0)	A	SER23	OG		4K1E	A	GLU165	OE 2		3.01
4K1E	A	SER169	OG		#2(-X,Y+½,-Z) + (0, 0, 0)	A	HIS25	O		3.418
4K1E	A	LYS170	NZ		#2(-X,Y+½,-Z) + (0, 0, 0)	A	ASP116	OD 1		2.877
#2(-X,Y+½,-Z) + (0, 0, 0)	A	SER135	OG		4K1E	A	ASN95	O	loop 4	2.752
4K1E	A	ARG150	NH 1	loop 5	#1(X,Y , Z) + (1, 0, 0)	A	SER111	OG		3.401
4K1E	A	ASN192	ND 2		#1(X,Y , Z) + (1, 0, 0)	A	GLN243	O		3.02
#1(X,Y , Z) + (1, 0, 0)	A	GLN243	NE 2		4K1E	A	CYS220	O	loop 8	3.449
4K1E	A	CYS220	N	loop 8	#1(X,Y , Z) + (1, 0, 0)	A	GLN243	OE 1		2.839
4K1E	A	LYS170	NZ		#1(X,Y , Z) + (1, 0, 1)	A	ASN61	O	loop 2	2.641
4K1E	A	LYS170	NZ		#1(X,Y , Z) + (1, 0, 1)	A	ASN61	OD 1	loop 2	3.32
4K1E	A	LYS170	NZ		#1(X,Y , Z) + (1, 0, 1)	A	SER86	O		2.617

A.

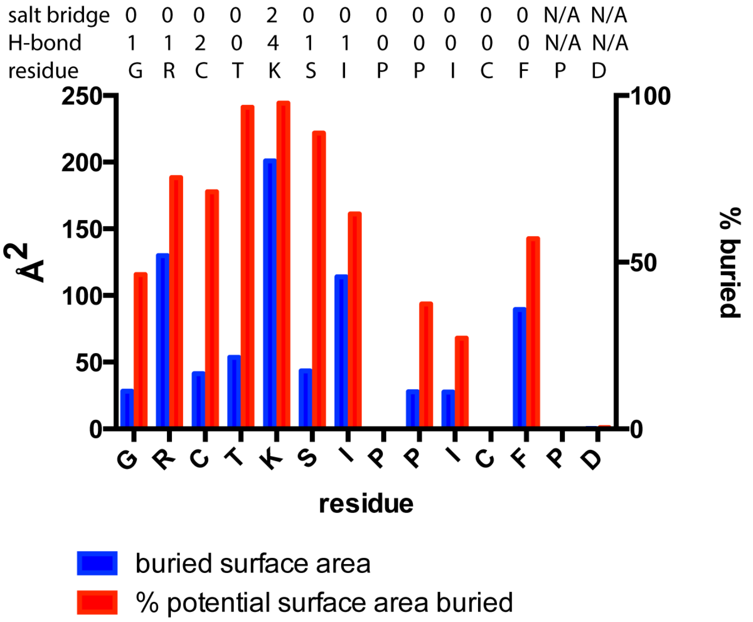
salt bridge 0 0 0 0 2 0 0 0 0 0 0 0 N/A N/A
H-bond 1 1 2 0 4 1 1 0 0 0 0 0 N/A N/A
residue G R C T K S I P P I C F P D
Å2
% buried
residue
buried surface area
% potential surface area buried

B.

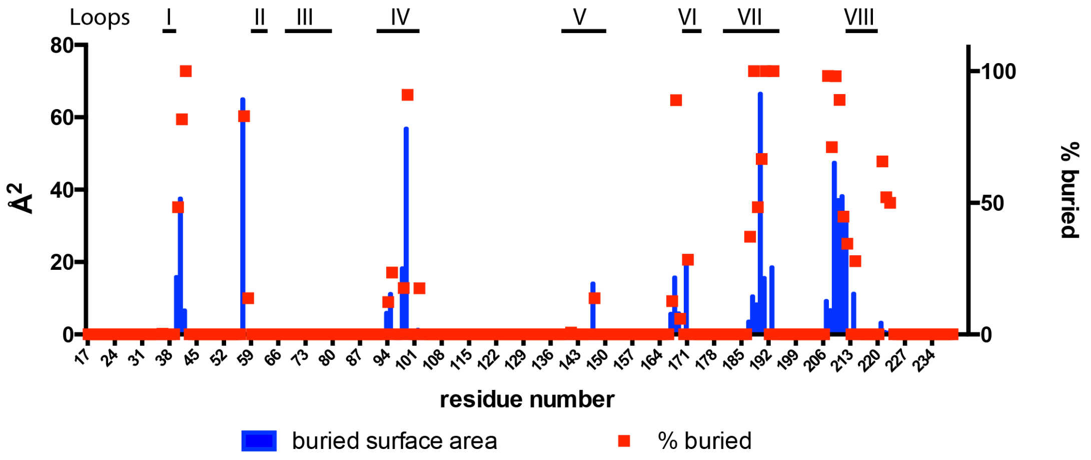
Loops I II III IV V VI VII VIII
Å2
% buried
residue number
buried surface area
% buried

C.

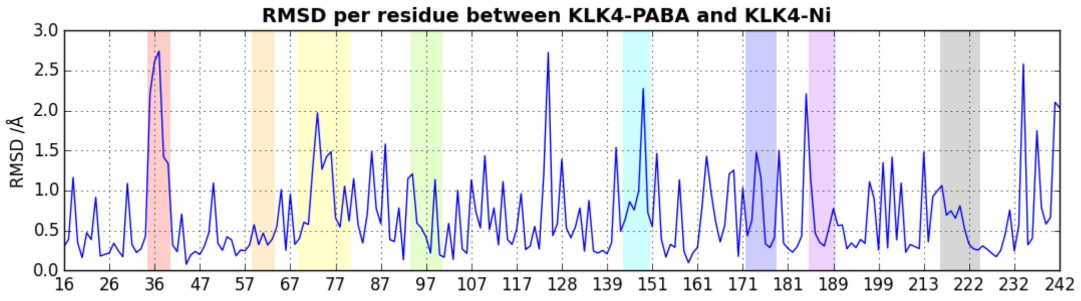
RMSD per residue between KLK4-PABA and KLK4-Ni
RMSD /Å

Fig. S1. Protein-inhibitor interaction data in KLK4-SFTI-1. **(A)** SFTI-1 residues engaged in salt bridges and H-bonds with KLK4 are indicated by the number of interactions at the top of Fig. S1A. The buried surface area per residue is indicated by blue bars (left Y-axis). The complementarity of the interaction is measured by the percent of accessible surface area that is buried (red bars, right Y-axis); **(B)** The KLK4 residues involved in the interaction with SFTI-1. KLK4 residues 16-239 are represented with their contributions to the buried surface area (left axis- blue bars) and the complementarity of each of the residues (determined by the percent of accessible surface area that is buried; right axis- red squares). The loop designations are shown by the bars on the top with Roman numerals I-VIII; **(C)** Structural changes of KLK4 upon binding SFTI-1 as shown by a plot of heavy-atom RMSD by residue after structural alignment of KLK4-PABA with KLK4-SFTI-1. The most variable loop I is highly variable within the A & B chains of single structures and is not correlated with changes in ligand occupancy.

A

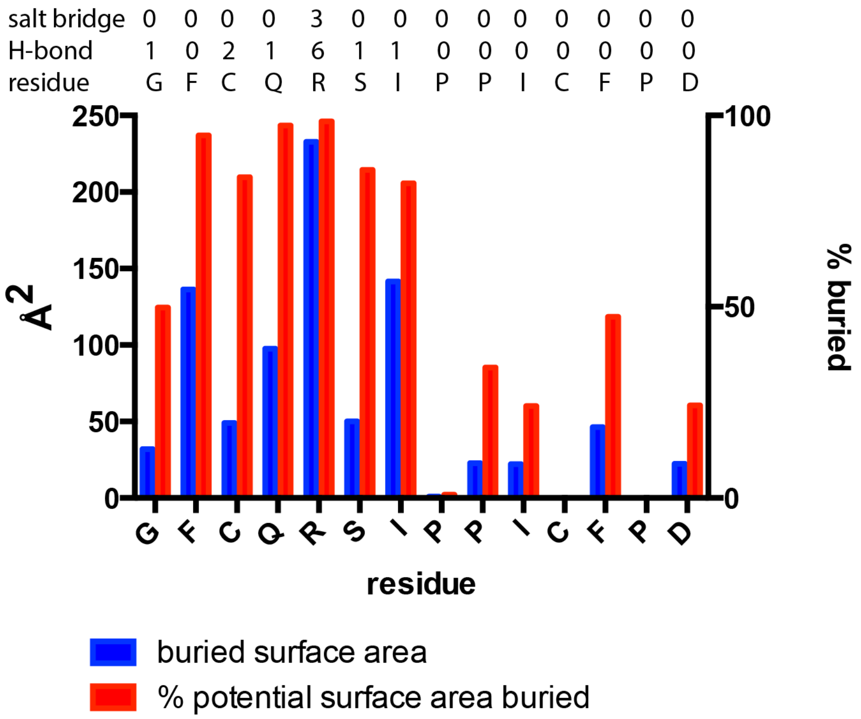

B

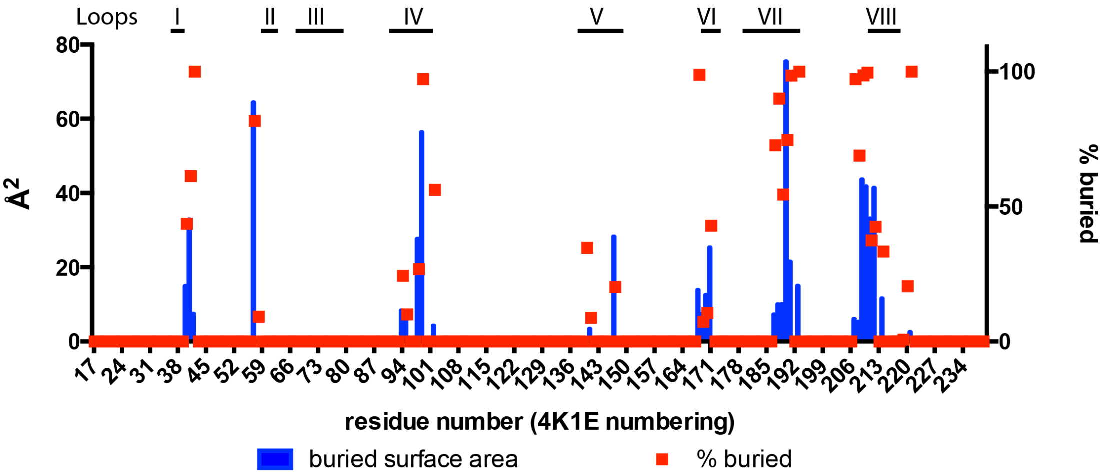

Fig. S2. Protein-inhibitor interactions for KLK4-SFTI-1$_{FCQR}$. **(A)** SFTI-1$_{FCQR}$ residues engaged in salt bridges and H-bonds with KLK4 are indicated by the number of interactions at the top of Fig. S1A. The buried surface area per residue is shown by blue bars (left Y-axis). The complementarity of the interaction is measured by the percent of accessible surface area that is buried (red bars, right Y-axis); **(B)** KLK4 residues involved in the interaction with SFTI-1$_{FCQR}$. KLK4 residues 16-239 are represented with their contributions to the buried surface area (left axis - blue bars) and the complementarity of each of the residues (determined by the percentage accessible surface area buried; right

axis - red squares). The loop designations are shown by the bars on the top with Roman numerals I-VIII.

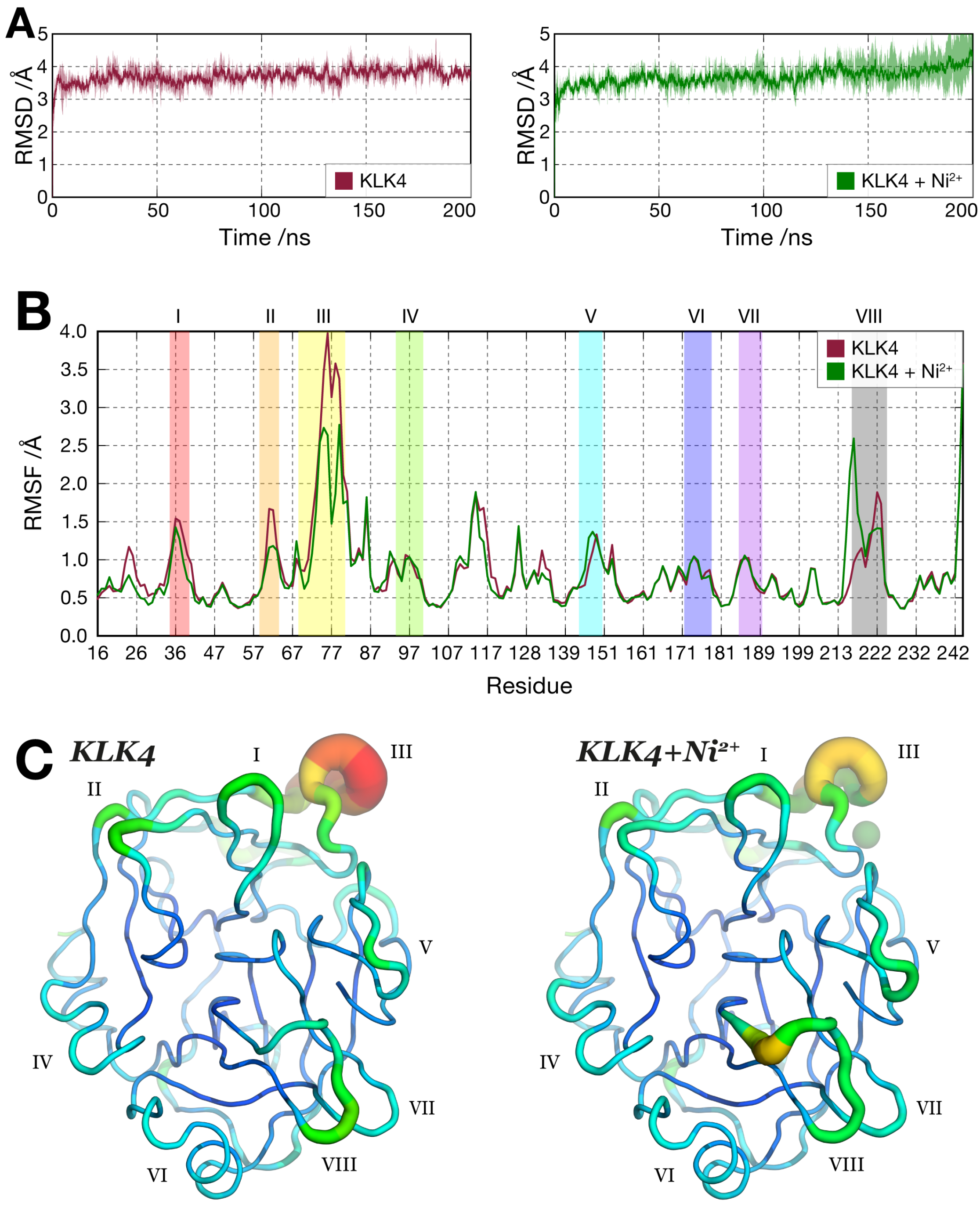

Fig. S3. Molecular dynamics simulation of KLK4-apo and KLK4-Ni. (A) Root mean square deviation (RMSD) plots of Cα atoms in KLK4-apo and KLK4-Ni over 200 ns at 300 K. Plots show the mean RMSD (solid line) with the min/max variation (n=3); (B) Root mean square fluctuation (RMSF) plots of Cα atoms in KLK4-apo and KLK4-Ni over 200 ns at 300 K (n=3); (C) KLK4-apo (left) and KLK4-Ni shown with RMSF values represented as colored putty (blue & thin = low, green = medium, red & thick = high).

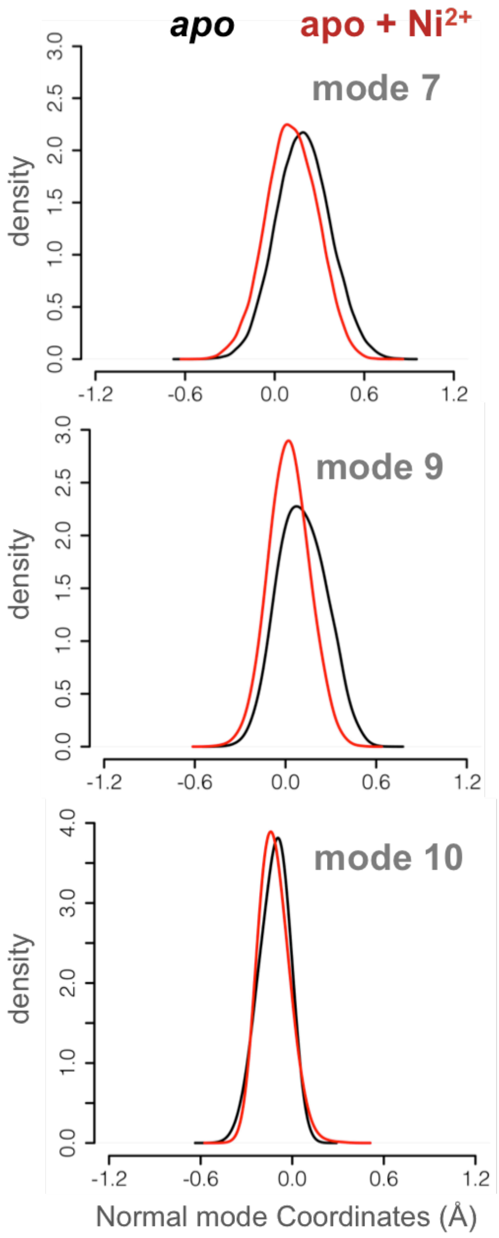

Fig. S4: Distributions of NM coordinates calculated from conformational states sampled during MD trajectories (KLK4-apo = black; KLK4-Ni = red) for other low frequency modes. The high similarity between the curves show that such motions are not affected by the Ni^{2+} interaction.

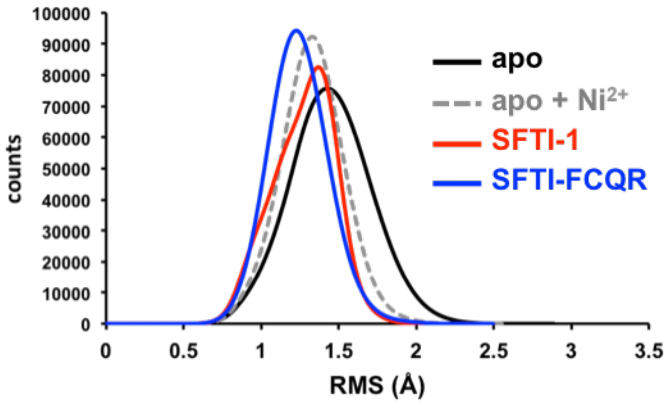

Fig. S5: Distributions of pairwise RMSD values calculated from each trajectory considering KLK4 C-alpha atoms. The width of the distributions is related to the size of the conformational spaces sampled. The narrow distribution centered at a low RMS value (1.2 Å) obtained in the presence of SFTI-1_{FCQR} (blue curve) shows the improved ability of the inhibitor to restrict the intrinsic KLK4 structural variability. The color-code is indicated in the legend.

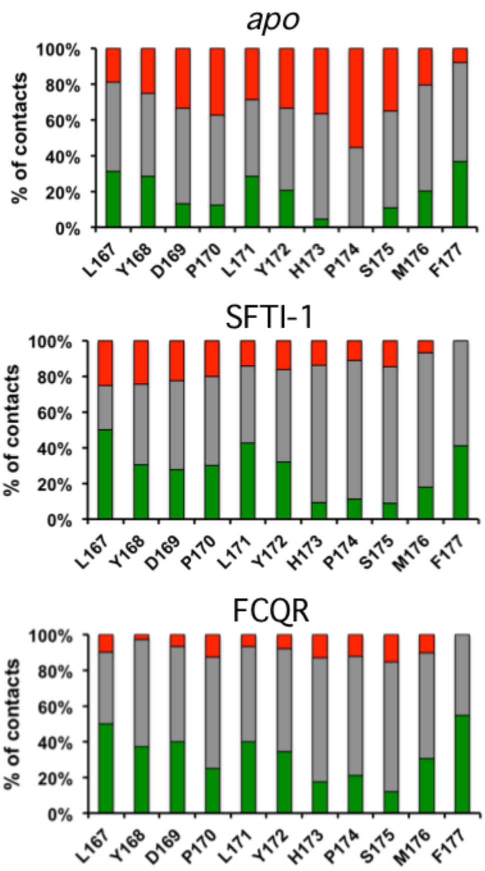

Fig. S6: Fraction of frustrated contacts per loop VI residue (*top*: apo; *middle*: KLK4-SFTI-1; *bottom*: KLK4-SFTI-1_{FCQR}). Minimally, neutral and highly frustrated contacts are represented in green, gray and red respectively.

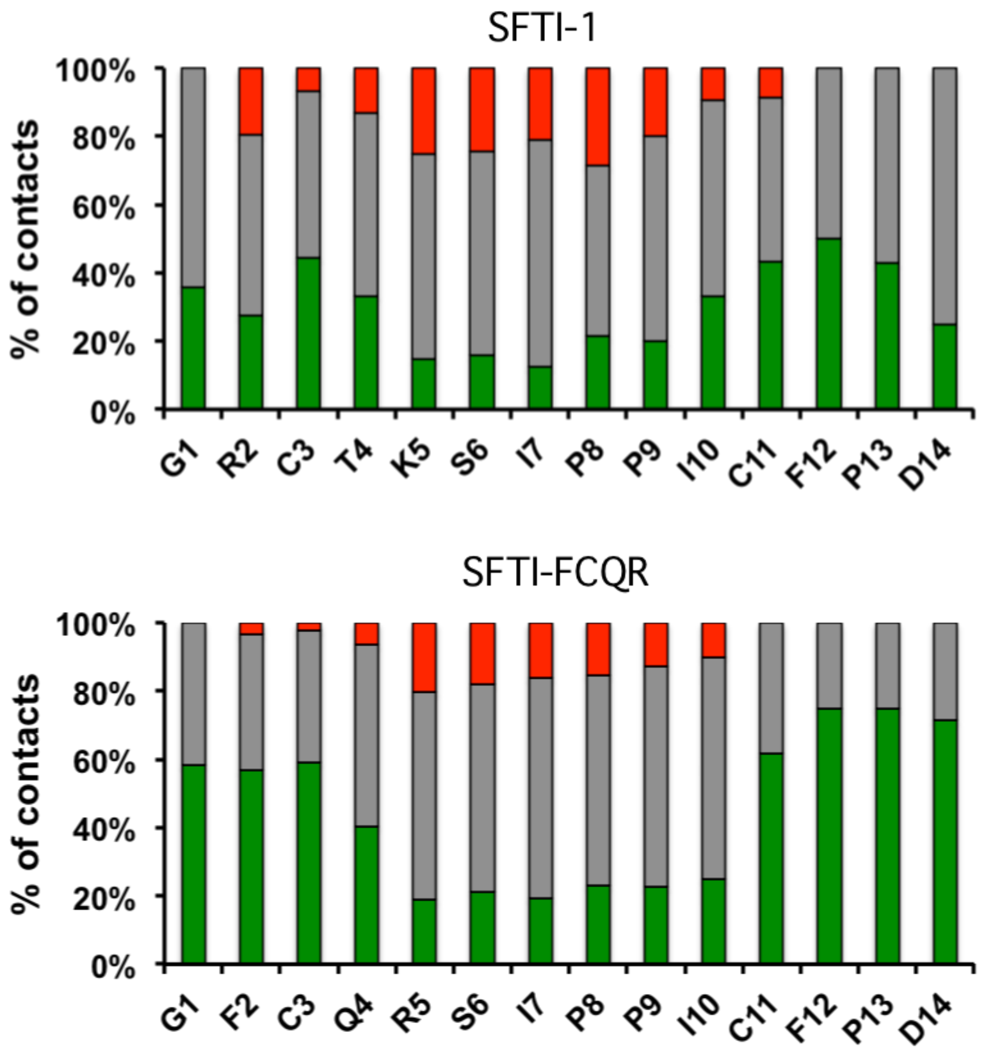

Fig. S7: Fraction of frustrated contacts per inhibitor position (*top*: SFTI-1; *bottom*: SFTI-1_{FCQR}). Minimally, neutral and highly frustrated contacts are represented in green, gray and red respectively. SFTI-1_{FCQR} presents the largest proportion of minimally frustrated interactions in almost all positions. In contrast, there are more highly frustrated contacts in SFTI-1 at positions 2-11.

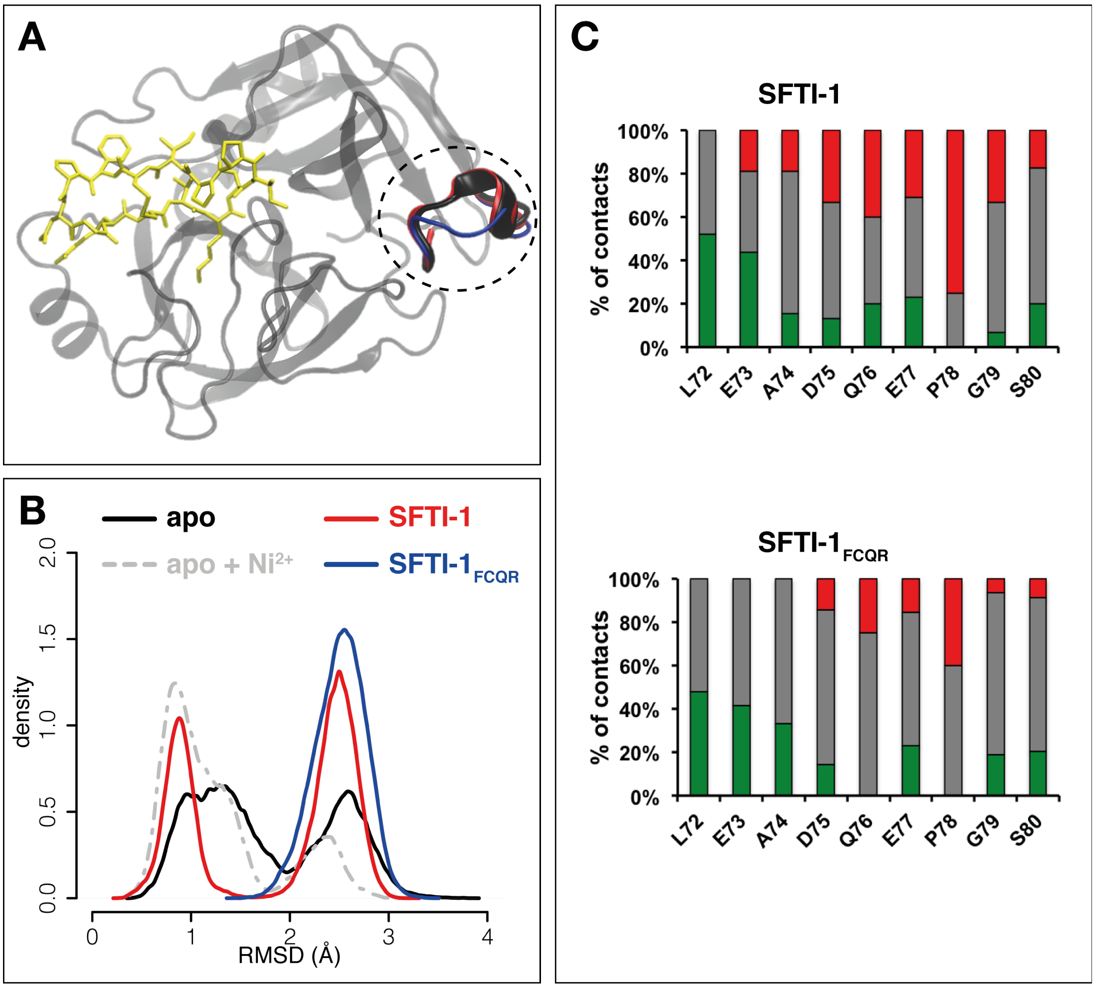

Fig. S8. Investigating allosteric modulation upon inhibitor binding. **(A)** Structural superposition between different KLK4 structures highlighting the loop III conformation. Black, red and blue correspond to *apo*, SFTI-1 and SFTI-1$_{FCQR}$ bound respectively. The SFTI structure is represented in sticks to facilitate the identification of the active site; **(B)** Distribution of loop III RMSD values computed for each system; **(C)** Fraction of frustrated contacts per residue in each conformation. Minimally, neutral and highly frustrated contacts are represented in green, gray and red, respectively.

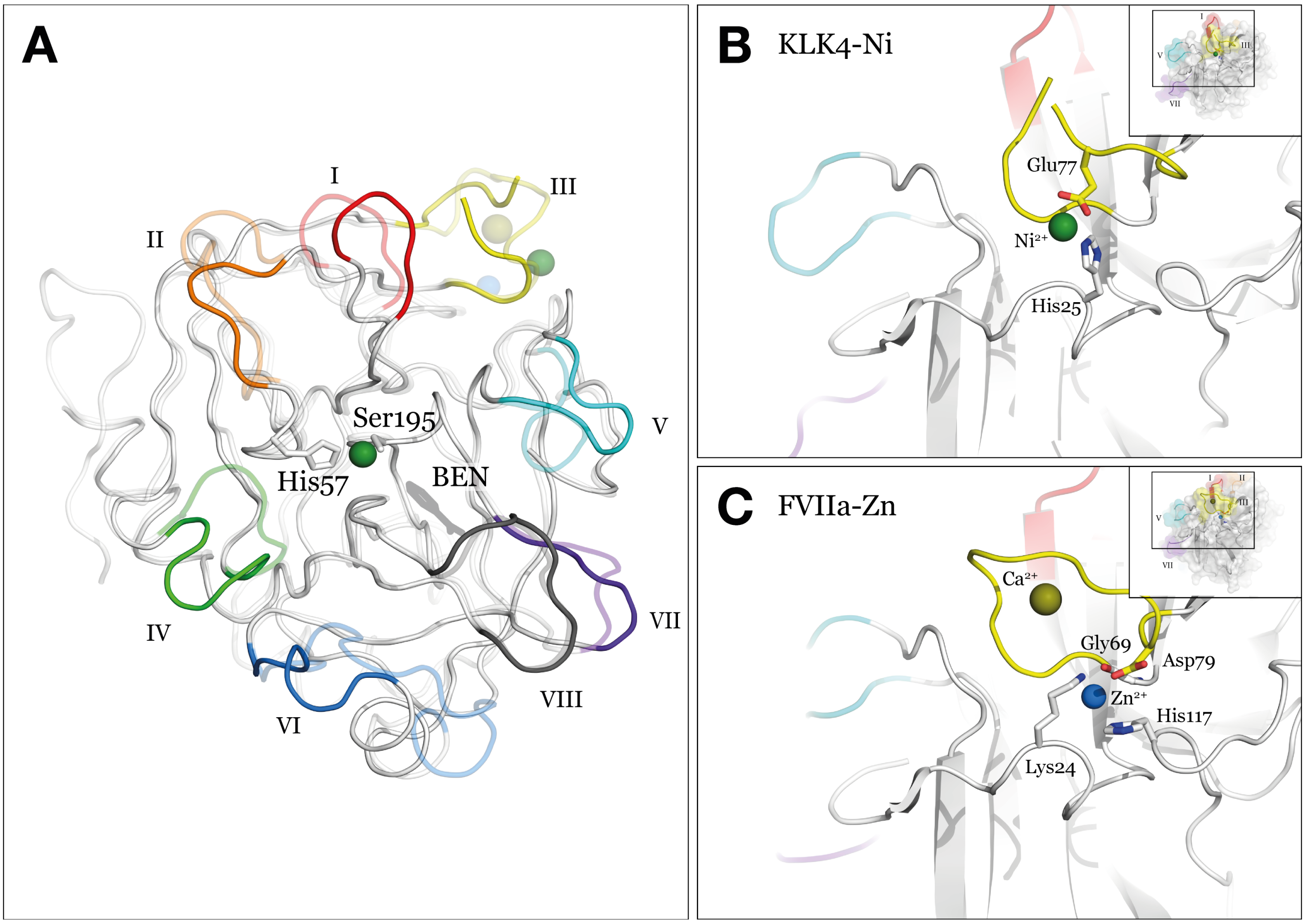

Fig. S9. A metal binding site in FVIIa is structurally similar to the His25/Glu77 site in KLK4. The KLK4-Ni structure (solid) superposed with the FVIIa serine protease domain (transparent; 2AER). A structural comparison shows that the FVIIa serine protease domain has one zinc site that aligns well with the Ni/Zn His25/Glu77 site in KLK4. This “site-2” FVIIa zinc is within 3.6 Å of the KLK4 metal binding site in the compared structures. The amino acids that chelate the metal are quite different to their KLK4 counterparts, with FVIIa using Lys24, Gly69 (loop III), Asp79 (loop III) and His117. However, the theme is the same: a metal bridge between the N-terminal strand (Lys24) and loop III. FVIIa also has a calcium ion that is chelated by loop III. The nickel bound to KLK4 His25/Glu77 is in green. The zinc atom bound to factor VIIa “site-2” is in blue, while the calcium bound to factor VIIa loop III is in gold.

Supplemental movie S1. A representative example of the dynamics of residues FGK during MD simulations. A static SFTI-1 structure has been superimposed into the active site at the beginning of the simulations for context. Inhibitory motions with respect to SFTI-1 can then be easily monitored throughout the simulation.

Supplemental movie S2. 2 Å displacement along normal mode 8

SI References

1 Takayama, T. K., McMullen, B. A., Nelson, P. S., Matsumura, M. & Fujikawa, K. Characterization of hK4 (prostase), a prostate-specific serine protease: activation of the precursor of prostate specific antigen (pro-PSA) and single-chain urokinase-type plasminogen activator and degradation of prostatic acid phosphatase. *Biochemistry* **40**, 15341-15348 (2001).

2 Swedberg, J. E. *et al.* Substrate-guided design of a potent and selective kallikrein-related peptidase inhibitor for kallikrein 4. *Chemistry & biology* **16**, 633-643, doi:10.1016/j.chembiol.2009.05.008 (2009).

3 McPhillips, T. M. *et al.* Blu-Ice and the Distributed Control System: software for data acquisition and instrument control at macromolecular crystallography beamlines. *Journal of synchrotron radiation* **9**, 401-406 (2002).

4 Cowieson, N. P. *et al.* MX1: a bending-magnet crystallography beamline serving both chemical and macromolecular crystallography communities at the Australian Synchrotron. *Journal of synchrotron radiation* **22**, 187-190, doi:10.1107/S1600577514021717 (2015).

5 Evans, P. Scaling and assessment of data quality. *Acta crystallographica. Section D, Biological crystallography* **62**, 72-82, doi:10.1107/S0907444905036693 (2006).

6 Winn, M. D. *et al.* Overview of the CCP4 suite and current developments. *Acta crystallographica. Section D, Biological crystallography* **67**, 235-242, doi:10.1107/S0907444910045749 (2011).

7 Battye, T. G., Kontogiannis, L., Johnson, O., Powell, H. R. & Leslie, A. G. iMOSFLM: a new graphical interface for diffraction-image processing with MOSFLM. *Acta crystallographica. Section D, Biological crystallography* **67**, 271-281, doi:10.1107/S0907444910048675 (2011).

8 McCoy, A. J. *et al.* Phaser crystallographic software. *Journal of applied crystallography* **40**, 658-674, doi:10.1107/S0021889807021206 (2007).

9 Debela, M. *et al.* Crystal structures of human tissue kallikrein 4: activity modulation by a specific zinc binding site. *Journal of molecular biology* **362**, 1094-1107, doi:10.1016/j.jmb.2006.08.003 (2006).

10 Adams, P. D. *et al.* PHENIX: a comprehensive Python-based system for macromolecular structure solution. *Acta crystallographica. Section D, Biological crystallography* **66**, 213-221, doi:10.1107/S0907444909052925 (2010).

11 Emsley, P. & Cowtan, K. Coot: model-building tools for molecular graphics. *Acta crystallographica. Section D, Biological crystallography* **60**, 2126-2132, doi:10.1107/S0907444904019158 (2004).

12 Dolinsky, T. J., Nielsen, J. E., McCammon, J. A. & Baker, N. A. PDB2PQR: an automated pipeline for the setup of Poisson–Boltzmann electrostatics calculations. *Nucleic acids research* **32**, W665-W667 (2004).

13 Søndergaard, C. R., Olsson, M. H., Rostkowski, M. & Jensen, J. H. Improved treatment of ligands and coupling effects in empirical calculation and rationalization of p K a values. *Journal of Chemical Theory and Computation* **7**, 2284-2295 (2011).

14 Joung, I. S. & Cheatham, T. E. Determination of Alkali and Halide Monovalent Ion Parameters for Use in Explicitly Solvated Biomolecular Simulations. *The Journal of Physical Chemistry B* **112**, 9020-9041 (2008).

15 Maier, J. A. *et al.* ff14SB: Improving the Accuracy of Protein Side Chain and Backbone Parameters from ff99SB. *Journal of Chemical Theory and Computation* **11**, 3696-3713 (2015).
16 Li, P., Roberts, B. P., Chakravorty, D. K. & Merz Jr, K. M. Rational design of particle mesh Ewald compatible Lennard-Jones parameters for +2 metal cations in explicit solvent. *Journal of chemical theory and computation* **9**, 2733-2748 (2013).
17 Jorgensen, W. L., Chandrasekhar, J., Madura, J. D., Impey, R. W. & Klein, M. L. Comparison of simple potential functions for simulating liquid water. *The Journal of Chemical Physics* **79**, 926-935, doi:doi:http://dx.doi.org/10.1063/1.445869 (1983).
18 Berendsen, H. J. C., Postma, J. P. M., van Gunsteren, W. F., DiNola, A. & Haak, J. R. Molecular dynamics with coupling to an external bath. *The Journal of Chemical Physics* **81** (1984).
19 Phillips, J. C. *et al.* Scalable molecular dynamics with NAMD. *Journal of computational chemistry* **26**, 1781-1802 (2005).
20 Darden, T., York, D. & Pedersen, L. Particle mesh Ewald: An N·log(N) method for Ewald sums in large systems. *The Journal of Chemical Physics* **98**, 10089-10092, doi:doi:http://dx.doi.org/10.1063/1.464397 (1993).
21 Brooks, B. R. *et al.* CHARMM: the biomolecular simulation program. *Journal of computational chemistry* **30**, 1545-1614, doi:10.1002/jcc.21287 (2009).
22 Hornak, V. *et al.* Comparison of multiple Amber force fields and development of improved protein backbone parameters. *Proteins* **65**, 712-725, doi:10.1002/prot.21123 (2006).
23 Krissinel, E. & Henrick, K. Inference of macromolecular assemblies from crystalline state. *Journal of molecular biology* **372**, 774-797, doi:10.1016/j.jmb.2007.05.022 (2007).
